# *Candida albicans* Ethanol Stimulates *Pseudomonas aeruginosa* WspR-Controlled Biofilm Formation as Part of a Cyclic Relationship Involving Phenazines

**DOI:** 10.1371/journal.ppat.1004480

**Published:** 2014-10-23

**Authors:** Annie I. Chen, Emily F. Dolben, Chinweike Okegbe, Colleen E. Harty, Yuriy Golub, Sandy Thao, Dae Gon Ha, Sven D. Willger, George A. O'Toole, Caroline S. Harwood, Lars E. P. Dietrich, Deborah A. Hogan

**Affiliations:** 1 Department of Microbiology and Immunology, Geisel School of Medicine at Dartmouth, Hanover, New Hampshire, United States of America; 2 Department of Biological Sciences, Columbia University, New York, New York, United States of America; 3 Department of Microbiology, School of Medicine, University of Washington, Seattle, Washington, United States of America; Yale University, United States of America

## Abstract

In chronic infections, pathogens are often in the presence of other microbial species. For example, *Pseudomonas aeruginosa* is a common and detrimental lung pathogen in individuals with cystic fibrosis (CF) and co-infections with *Candida albicans* are common. Here, we show that *P. aeruginosa* biofilm formation and phenazine production were strongly influenced by ethanol produced by the fungus *C. albicans*. Ethanol stimulated phenotypes that are indicative of increased levels of cyclic-di-GMP (c-di-GMP), and levels of c-di-GMP were 2-fold higher in the presence of ethanol. Through a genetic screen, we found that the diguanylate cyclase WspR was required for ethanol stimulation of c-di-GMP. Multiple lines of evidence indicate that ethanol stimulates WspR signaling through its cognate sensor WspA, and promotes WspR-dependent activation of Pel exopolysaccharide production, which contributes to biofilm maturation. We also found that ethanol stimulation of WspR promoted *P. aeruginosa* colonization of CF airway epithelial cells. *P. aeruginosa* production of phenazines occurs both in the CF lung and in culture, and phenazines enhance ethanol production by *C. albicans*. Using a *C. albicans adh1*/*adh1* mutant with decreased ethanol production, we found that fungal ethanol strongly altered the spectrum of *P. aeruginosa* phenazines in favor of those that are most effective against fungi. Thus, a feedback cycle comprised of ethanol and phenazines drives this polymicrobial interaction, and these relationships may provide insight into why co-infection with both *P. aeruginosa* and *C. albicans* has been associated with worse outcomes in cystic fibrosis.

## Author Summary

In many human infections, several species of microbes are often present. This is typically the case with the disease cystic fibrosis, characterized by thick mucus in the lungs that is colonized by bacteria and fungi. Here, we show evidence that interactions between the bacterium *Pseudomonas aeruginosa* and the fungus *Candida albicans* result in attributes of infection that are worse for the human host. We found that ethanol, such as that produced by *C. albicans*, causes increased levels of a signaling molecule in *P. aeruginosa* that promotes biofilm formation. Biofilm formation by *P. aeruginosa* is associated with infections that are more difficult to treat. Ethanol stimulated *P. aeruginosa* colonization of plastic surfaces and airway cells, and we identified components of this mechanism. Fungally-produced ethanol also changes the spectrum of phenazine toxins produced by *P. aeruginosa*, and phenazines are associated with worse lung function in people with cystic fibrosis. In light of the fact that phenazines interact with *C. albicans* to promote ethanol production, we propose a positive feedback loop between *C. albicans* and *P. aeruginosa* that contributes to worse disease. Our findings could have implications for the study and treatment of multi-species infections.

## Introduction

*Pseudomonas aeruginosa* is an opportunistic pathogen capable of causing severe nosocomial infections and infections in immunocompromised patients. *P. aeruginosa* is a common pathogen of individuals with cystic fibrosis (CF), a genetic disease that is caused by a mutation in the gene coding for the CFTR ion transporter and strongly associated with chronic, recalcitrant lung infections. Altered CFTR function leads to a fluid imbalance that results in thick, sticky mucus in the lungs that is difficult to clear, thus creating a hospitable environment for microbial growth, biofilm formation, and persistence. While *P. aeruginosa* is a common microbe in the CF lung, it is rarely the only microbe present [1]–[5]. Co-infections of *P. aeruginosa* with other bacterial and fungal species are common, and there is a need to understand how these complex multi-species infections impact disease course and treatability. For example, the presence of the fungus *Candida albicans* correlates with more frequent exacerbations and a more rapid loss of lung function in CF patients [6], [7]. Additional studies are needed to determine if the presence of the fungus contributes to more severe disease.

Published reports strongly suggest that in the CF lung, *P. aeruginosa* forms biofilms [8], described as hearty aggregations of cells in a sessile group lifestyle that includes extracellular matrix comprised of proteins, membrane vesicles, DNA, and exopolysaccharides. A biofilm existence provides many advantages to *P. aeruginosa* including increased antibiotic tolerance [9], [10]. As with many Gram-negative species, *P. aeruginosa* biofilm formation is positively regulated by the secondary signaling molecule cyclic-di-GMP (c-di-GMP) [11]. C-di-GMP is formed from two molecules of GTP by diguanylate cyclases (DGCs) and its levels inversely correlate to motility. High levels of c-di-GMP promote biofilm formation in a number of ways including via increased matrix production and decreased flagellar motility [12]–[14].

*P. aeruginosa* also produces a class of redox-active virulence factors called phenazines. In CF sputum, the phenazines pyocyanin (PYO) and phenazine-1-carboxylate (PCA) are found in micromolar (5–80 µM) concentrations, and their levels are inversely correlated with lung function [15]. Phenazines play a role in the relationships between *P. aeruginosa* and eukaryotic cells. Several studies have shown how phenazines can negatively affect mammalian physiology [16], [17]. In addition, phenazines impact different fungi, including *C. albicans*. At high concentrations, phenazines are toxic to *C. albicans*, and lower concentrations of phenazines reduce fungal respiration and impair growth as hyphae [18]. Phenazines figure prominently in shaping the chemical ecology within mixed-species communities. For example, when exposed to low concentrations of phenazines, *C. albicans* increases the production of fermentation products such as ethanol by 3 to 5 fold [18]. Furthermore, *P. aeruginosa*-*C. albicans* co-cultures form red derivatives of 5-methyl-phenazine-1-carboxylic acid (5MPCA) that accumulate within fungal cells [19].

In the present study, we show that ethanol produced by *C. albicans* stimulated *P. aeruginosa* biofilm formation and altered phenazine production. Ethanol caused a decrease in surface motility in both strains PA14 and PAO1 concomitant with a stimulation in levels of c-di-GMP, a second messenger nucleotide that promotes biofilm formation. Through a genetic screen, we found that the diguanylate cyclase WspR, a response regulator of the Wsp chemosensory system, was required for this response. Elements upstream and downstream of WspR signaling were required for the ethanol response. Ethanol no longer stimulated biofilm formation in a mutant lacking WspA, the membrane-localized sensor methyl-accepting chemotaxis protein (MCP) that is involved in the activation of WspR [20]. In addition, an intact Pel exopolysaccharide biosynthesis pathway, known to be stimulated by c-di-GMP derived from the Wsp pathway [21], [22], was also required for ethanol stimulation of biofilm formation. The effects were observed on both abiotic surfaces and a cell culture model for *P. aeruginosa* and *P. aeruginosa-C. albicans* airway colonization. We found that both exogenous and fungally-produced ethanol enhanced the production of two phenazine derivatives known for their antifungal activity [19], [23], 5MPCA and phenazine-1-carboxamide (PCN), through a Wsp-independent pathway and independent of ethanol catabolism. Because phenazines stimulate fungal ethanol production [18], we present evidence for a signaling cycle that helps drive this polymicrobial interaction.

## Results

### Ethanol stimulates biofilm formation and suppresses swarming in *P. aeruginosa* strain PA14

Our previously reported findings show that *P. aeruginosa* produces higher levels of two phenazines, PYO and 5MPCA [24], [25], when cultured with *C. albicans* and that phenazines stimulate *C. albicans* ethanol production [18]. Thus, we sought to determine how fungally-derived ethanol affects *P. aeruginosa*. A concentration of 1% ethanol (v/v) was chosen for these studies based on the detection of comparable levels of ethanol in *C. albicans* supernatants from cultures grown with phenazines [18]. The presence of 1% ethanol in the culture medium did not affect *P. aeruginosa* growth in minimal M63 medium with glucose (Fig. S1) or LB (doubling time of 36±2 min in LB versus 39±2 min in LB with ethanol), or on solid LB medium (Fig. S1 inset) except that the final culture yield in M63 was slightly higher in cultures amended with ethanol (Fig. S1).

**Figure 1:**
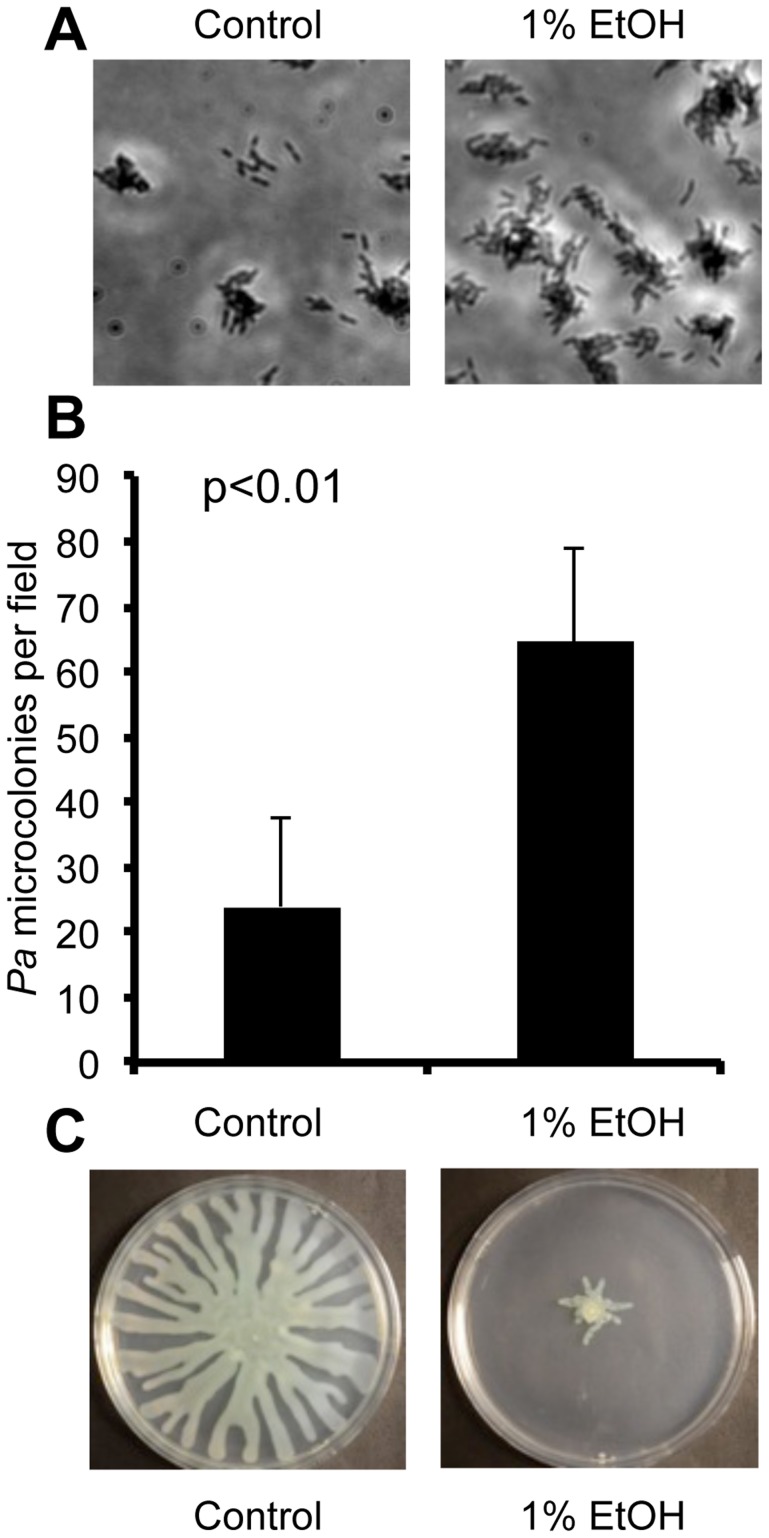
Ethanol represses swarming and stimulates biofilm formation by *P. aeruginosa*. **A**. *P. aeruginosa* strain PAO1 attachment to the bottom of a polystyrene plastic well after 6 hours in medium with and without 1% ethanol (EtOH). **B**. *P. aeruginosa* strain PA14 attachment to plastic as assessed by quantification of microcolonies per field in wells containing medium with or without ethanol for 7 h. Error bars represent the standard deviation (p<0.01 as determined by a student's t-test, N = 12). **C**. *P. aeruginosa* strain PA14 swarming in the absence and presence of 1% ethanol. Images are representative of results in more than ten independent experiments.

When we performed a microscopic analysis of the effects of ethanol on *P. aeruginosa* strain PAO1, we observed a significant increase in attachment of cells to the bottom of a titer dish well within 1 h (15±5 cells per field in vehicle treated compared to 31±6 cells per field in cultures with ethanol, p<0.01) and development of microcolonies was strongly enhanced (Fig. 1A). Ethanol also promoted an increase in the number of attached cells and microcolonies in cultures of another *P. aeruginosa* strain, PA14 (Fig. 1B).

Using two assays that assess biofilm-related phenotypes (swarming motility and twitching motility), we sought to gain additional insight into how ethanol impacted biofilm formation. Our initial studies focused on strain PA14. We found that ethanol repressed swarming motility, a behavior that is inversely correlated with biofilm formation (Fig. 1C). Ethanol did not affect type IV-pili-dependent twitching motility, a form of movement that is required for microcolony formation in biofilms on plastic (Fig. S2B) [26].

### Ethanol catabolism is not necessary for the inhibition of swarming

Because *P. aeruginosa* can catabolize ethanol [27], we sought to determine if ethanol consumption contributed to the repression of swarming motility. *P. aeruginosa* first oxidizes ethanol to acetaldehyde by an ethanol dehydrogenase, ExaA, which requires the cofactor PQQ (pyrroloquinoline quinone) [27]. Acetaldehyde is further oxidized to acetate by an NAD+ dependent acetaldehyde dehydrogenase (ExaC), and the acetate is subsequently oxidized to acetyl-CoA by AcsA [28]. We retrieved the *exaA*::Tn*M*, *pqqB*::Tn*M*, and *acsA*::Tn*M* mutants predicted to be defective in ethanol catabolism from the *P. aeruginosa* strain PA14 NR transposon library [29] and confirmed the transposon insertion sites by PCR (see Materials and Methods for more detail). As predicted, none of these mutants grew with ethanol as the sole carbon source, and growth on glucose was unaffected (Fig. S3A).

When we used these mutants in the swarm assay, we found no difference in the effects of ethanol on these three ethanol catabolism mutants in comparison to the wild-type parental strain (Fig. S3B) indicating that ethanol catabolism was not required for the ethanol response. Furthermore, other carbon sources such as glycerol, another fungal fermentation product, or choline, another two-carbon alcohol degraded by a PQQ-dependent enzyme, did not inhibit swarming motility (Fig. S4).

### Ethanol increases c-di-GMP levels through WspR

**Figure 2:**
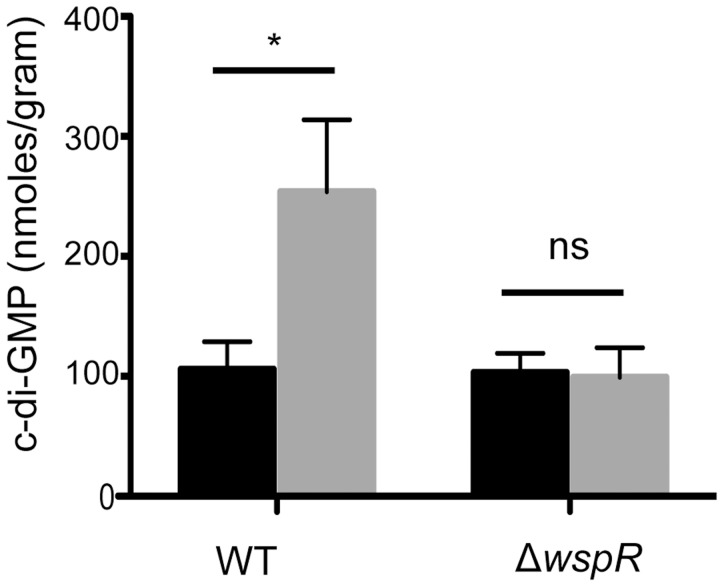
Ethanol increases c-di-GMP levels in *P. aeruginosa* strain PA14 WT but not in a Δ*wspR* mutant. c-di-GMP levels from cultures grown on swarm plates without (black) or with 1% ethanol (grey) were measured by LC-MS. Error bars represent one standard deviation (*,p<0.05, N = 5); ns, not significant).

Ethanol stimulated attachment and biofilm formation on plastic and inhibited swarming motility (Fig. 1). These two phenotypes are positively and negatively regulated by levels of the second messenger molecule c-di-GMP [30]. Thus, we measured intracellular levels of this dinucleotide in *P. aeruginosa* strain PA14 cells grown on swarm plates with or without 1% ethanol for 16.5 h as described previously. We found a 2.4-fold increase in c-di-GMP levels in cells exposed to ethanol (Fig. 2).

**Figure 3:**
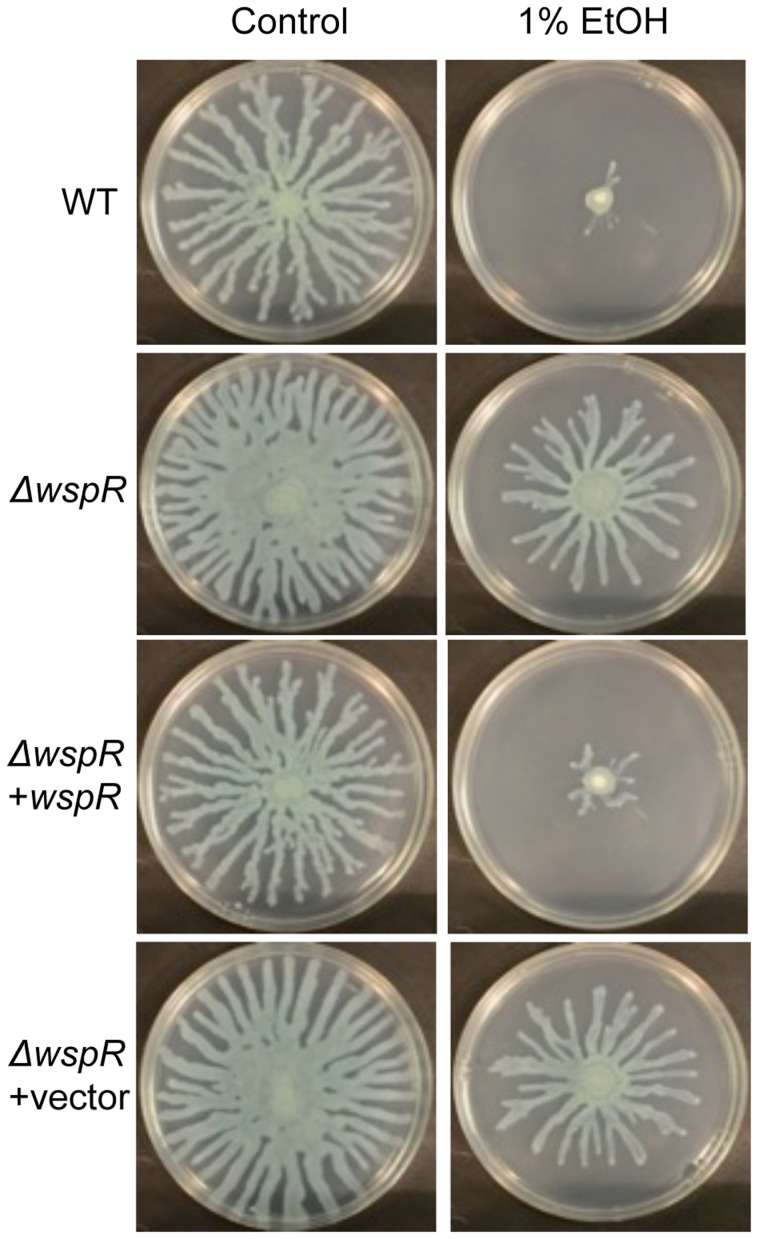
*P. aeruginosa* Δ*wspR* shows loss of swarm repression in the presence of ethanol. *P. aeruginosa* strain PA14 WT, Δ*wspR*, and Δ*wspR* strains containing either plasmid-borne *wspR* or the empty vector were analyzed on swarm medium with and without 1% ethanol (EtOH) and with 0.02% arabinose (to induce *wspR* expression in the complemented strain). Images are representative of at least 5 experiments for each strain.

To identify the enzyme(s) responsible for this increase, we screened a collection of 31 *P. aeruginosa* strain PA14 mutants [31] defective in different genes predicted to encode proteins that may modulate c-di-GMP levels based on the detection of a DGC and/or an EAL domain [22], [31]. We found that one mutant, Δ*wspR*, was strikingly resistant to the repression of swarming by ethanol (Fig. 3). As expected, this mutant also had a slight hyperswarming phenotype when compared to the wild type in control conditions [31], and both phenotypes were complemented by the wild-type *wspR* allele on an arabinose-inducible plasmid when grown in the presence of 0.02% arabinose (Fig. 3). The empty vector (EV) control exhibited a swarming pattern comparable to that of the Δ*wspR* mutant.

WspR is a response regulator with a GGDEF domain [32], which is associated with diguanylate cyclase activity [20]. Consistent with the observation that Δ*wspR* continued to swarm on medium with ethanol, c-di-GMP levels were not different between cultures with and without ethanol in the Δ*wspR* background (Fig. 2). These data suggest that WspR activity, and thus c-di-GMP levels, are enhanced by ethanol.

**Figure 4:**
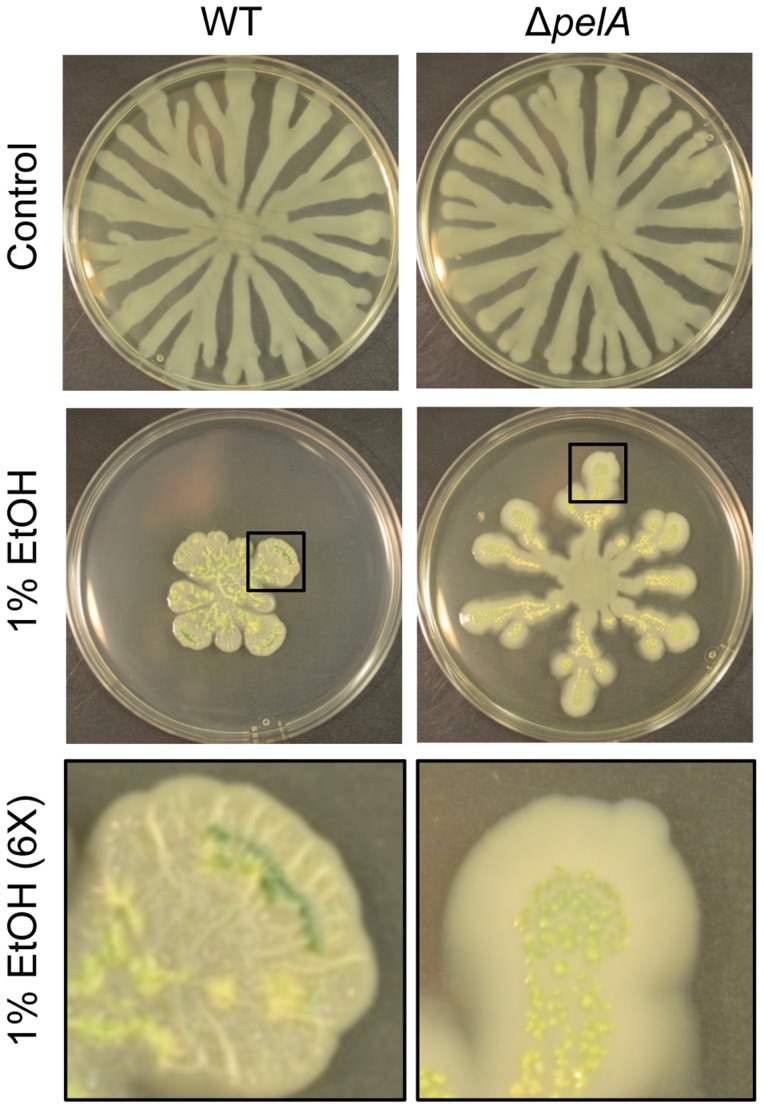
Pel production in response to ethanol. *P. aeruginosa* strain PA14 WT and Δ*pelA* swarm colonies on medium with and without 1% ethanol (EtOH) after 72 h. The bottom panels show an enlarged view (6×) of swarm tendrils of the colonies grown with ethanol (indicated by a black box). Enlarged images demonstrate the yellow/green PCN crystals in both strains and colony wrinkles in the WT that form upon growth on ethanol.

WspR is known to regulate the production of the Pel polysaccharide [21], [22], and production of Pel is associated with colony wrinkling and biofilm formation [33]. After 72 hours on swarm plates, we also observed that ethanol strongly promoted colony wrinkling while the addition of equivalent amounts of other carbon sources, such as glycerol or choline, did not have this effect. Furthermore, the colony wrinkling induced by ethanol was less apparent in a Δ*wspR* strain (not shown) and completely absent in a strain lacking *pelA*, an enzyme required for Pel biosynthesis (Fig. 4). The Δ*pelA* mutant, like the Δ*wspR* mutant, continued to swarm in the presence of ethanol (Fig. 4) suggesting that the repression of swarming in the presence of ethanol was, at least in part, due to increased Pel production.

### Ethanol induces c-di-GMP signaling in *P. aeruginosa* strain PAO1 through WspA and WspR

**Figure 5:**
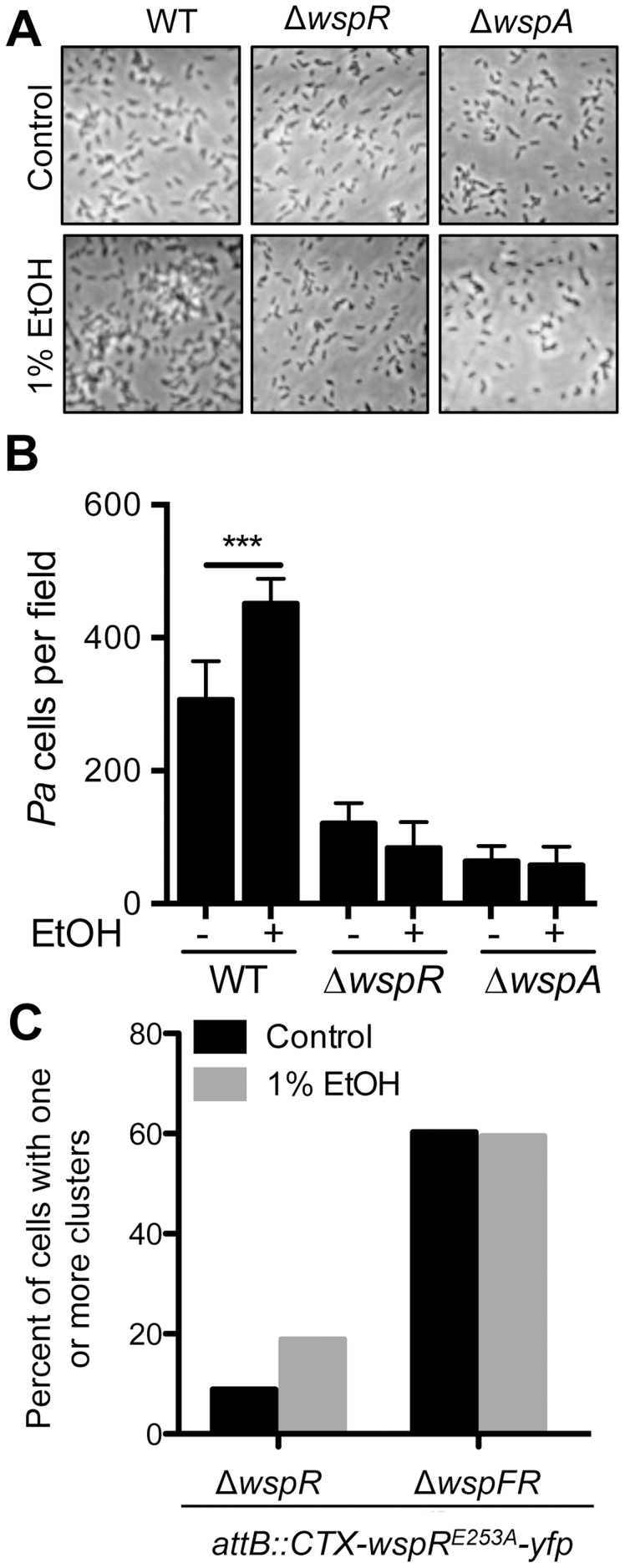
Ethanol acts through the Wsp system. **A**. Attachment of *P. aeruginosa* strain PAO1 WT, Δ*wspR* and Δ*wspA* mutants to the bottom of a polystyrene dish during growth in M63 medium with glucose and casamino acids, and with vehicle (Control) or with 1% ethanol for 6 hours. **B**. The number of cells per field for each condition was enumerated. Images and data are representative of results from more than three separate experiments. Significance determination was based on an ordinary one-way ANOVA followed by Sidak's multiple comparisons test for each intrastrain comparison; ***, *P*<0.001. **C**. Δ*wspR* and Δ*wspFR* strains expressing WspR-E253A-YFP were grown without and with 1% EtOH, and the number of cells with fluorescent clusters were counted out of a total of approximately 100 cells examined per condition across two experiments.

As we found that ethanol stimulated biofilm formation in *P. aeruginosa* wild-type strains PA14 and PAO1 (Fig. 1) and that WspR mediated the ethanol effect in strain PA14, we also examined the role of WspR in the ethanol response in strain PAO1. As shown above, PAO1 wild-type cells had increased early attachment and subsequent microcolony formation on plastic when ethanol was added to the medium (Fig. 5). Consistent with our model that ethanol is acting through WspR, ethanol did not stimulate surface colonization in the PAO1 Δ*wspR* mutant (Fig. 5). We also examined the ethanol-responsive phenotype for *P. aeruginosa* strain PAO1 Δ*wspA*, which lacks the membrane bound receptor that is the most upstream element described in the Wsp system [20]. Like Δ*wspR*, Δ*wspA* did not show increased attachment to plastic upon the addition of ethanol (Fig. 5) suggesting that both the MCP sensor and the WspR response regulator were required for the response to ethanol. Ethanol also promoted colony wrinkling in strain PAO1, as was observed in strain PA14, consistent with the prediction that increased WspR activity would lead to increased matrix production. Enhanced wrinkling with ethanol was shown most clearly for both strains in non-motile (*flgK*) mutants which formed colonies of similar size regardless of the presence of ethanol (Fig. S5). Because strain PAO1 WT does not swarm robustly in control conditions, the effects of ethanol on swarming in strain PAO1 were not quantified.

### Ethanol promotes WspR clustering and a functional Wsp system is required for this effect

Previous studies have shown that the fluorescently-tagged WspR protein forms intracellular clusters when in its active phosphorylated form upon incubation of cells on an agar surface, and cluster formation is positively correlated with WspR activity [20]. To complement the mutant analyses, we determined if ethanol also promoted WspR-YFP clustering, and if known components of the WspR activation system were required for WspR stimulation by ethanol. To facilitate these analyses, we used the WspR variant WspR^E253A^-YFP, which forms larger clusters that are more easily visualized [34]. In these studies, we observed a two fold increase in WspR clustering in the presence of ethanol (Fig. 5C). To determine if WspF, a methylesterase that negatively regulates WspR activity [21], was involved in the regulation of WspR in response to ethanol, we also assessed WspR clustering in a Δ*wspF* background where WspR is constitutively active. In Δ*wspF*, WspR clustering was higher than in the *wspF*+ reference strain, and WspR clustering was not further stimulated by ethanol, lending support for the model that ethanol was acting through the Wsp system and not through an independent pathway for WspR activation.

### *C. albicans* and ethanol promote airway epithelial cell monolayer colonization

**Figure 6:**
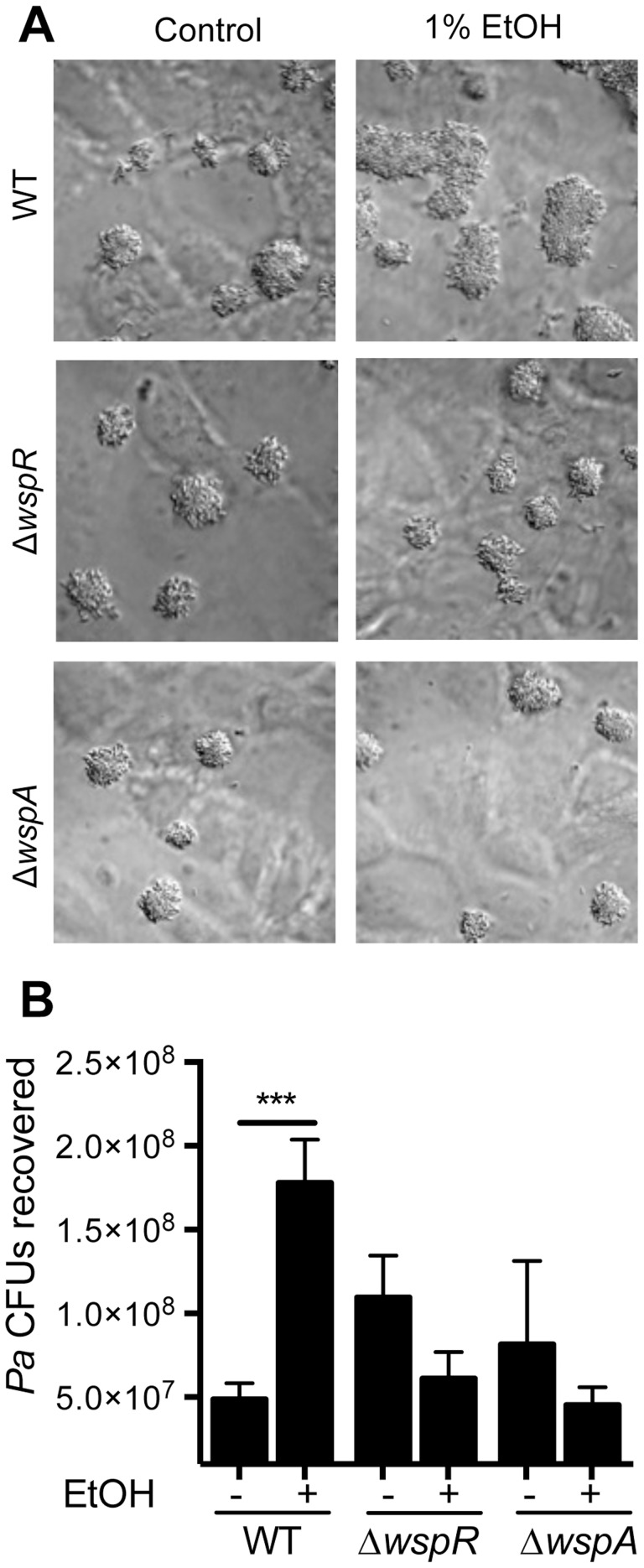
Ethanol significantly increases *P. aeruginosa* strain PAO1 WT biofilm formation on airway cells. **A**. PAO1 WT, Δ*wspR* or Δ*wspA* were co-cultured with a monolayer of ΔF508 CFTR-CFBE cells at an MOI of 30∶1 in medium with or without 1% ethanol and imaged after 6 h. Pictures are representative of at least 3 separate experiments with similar results. **B**. The number of CFUs from cultures determined as described above. Significance determination was based on an ordinary one-way ANOVA followed by Sidak's multiple comparisons test for each intrastrain comparison; ***, *P*<0.001. Error bars represent one standard deviation.

To understand the effects of ethanol on *P. aeruginosa* in a well-established CF-relevant disease model, we studied the effects of ethanol on *P. aeruginosa* strain PAO1 in the context of bronchial epithelial cells with the most common CF genotype (homozygous *CFTR*ΔF508) [35], [36]. We cultured *P. aeruginosa* strain PAO1 with the epithelial cells in medium without and with 1% ethanol, and observed an obvious enhancement in the size of biofilm microcolonies (Fig. 6A) and a 2.2-fold increase in colony forming units (CFUs) on the airway cells with ethanol (Fig. 6B). When the same experiment was performed with the Δ*wspR* or Δ*wspA* mutants, no stimulation by ethanol was observed. Ethanol alone did not impact epithelial cell viability as measured by an LDH release assay (9.44%±0.98 LDH release for control and 10.47%±1.2 LDH release with ethanol, N = 3) and other studies have also found these concentrations of ethanol to be well below those that cause overt toxicity to epithelial cells or disruption of epithelial barrier integrity [37], [38].

**Figure 7:**
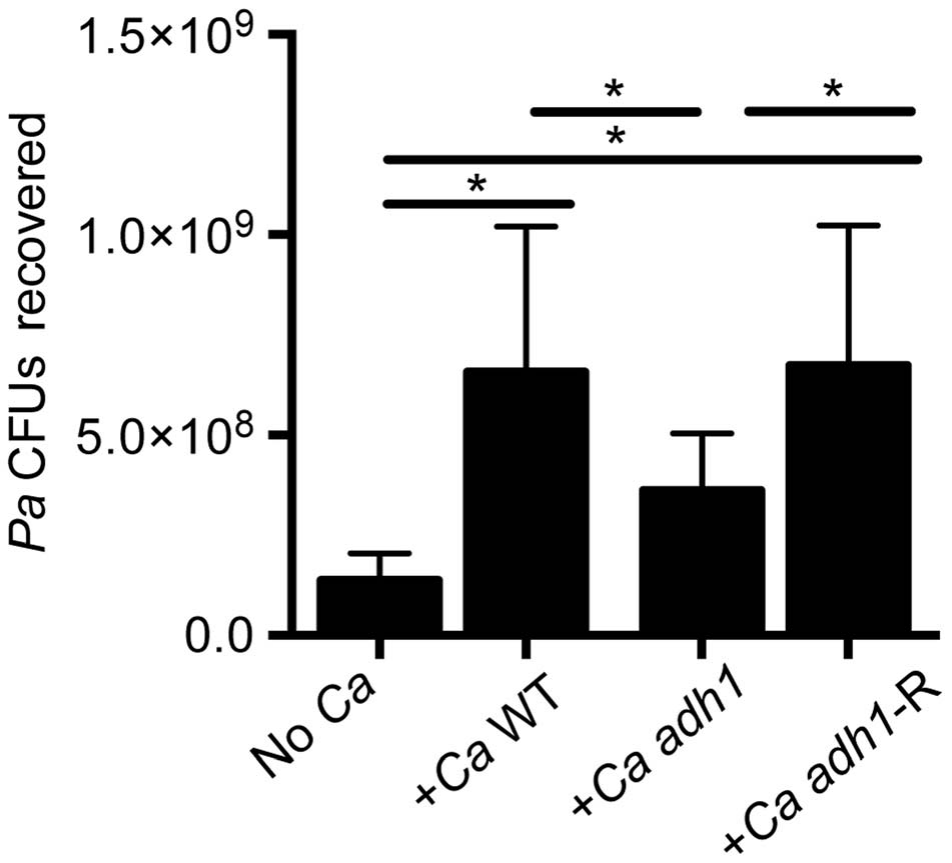
*C. albicans* promotes *P. aeruginosa* strain PAO1 WT biofilm formation on airway epithelial cells in part through ethanol production. *P. aeruginosa* PAO1 WT was cultured with a monolayer of ΔF508 CFTR-CFBE cells alone or with *C. albicans* CAF2 (reference strain), the *C. albicans adh1/adh1* mutant (*adh1*), and its complemented derivative, *adh1/adh1+ADH1* (*adh1-R*). Data are combined from three independent experiments with 3–5 technical replicates per experiment, (* represents a statistically significant difference (p<0.05) between indicated strains). Error bars represent one standard deviation.

When *P. aeruginosa* PAO1 and *C. albicans* were co-inoculated into epithelial cell co-cultures, 4.7-fold more *P. aeruginosa* CFUs were found to be associated with the monolayer after 6 h (Fig. 7). To determine if *C. albicans*-derived ethanol contributed to the enhanced colonization by *P. aeruginosa* in the presence of *C. albicans*, we used a *C. albicans adh1*/*adh1* mutant that produced lower levels of ethanol. We constructed the *adh1* null strain and its complemented derivative, and confirmed that the absence of *ADH1* caused a reduction in ethanol by HPLC analysis of culture supernatants, a finding consistent with previously published work [39]. When *P. aeruginosa* was co-cultured with the *C. albicans adh1*/*adh1* strain, there was a significant decrease in *P. aeruginosa* CFUs recovered, and this defect was corrected upon complementation with the *ADH1* gene in trans. Furthermore, there was no significant difference in the stimulation of colonization by wild-type or *adh1*/*adh1* mutant *C. albicans* in the Δ*wspR* or Δ*wspA* backgrounds (Fig. S6). Together, these data strongly suggest that *C. albicans*-produced ethanol promotes *P. aeruginosa* colonization of both abiotic and biotic surfaces through activation of the Wsp system, which likely exerts these effects through promoting Pel production.

### Exogenous and *C. albicans*-produced ethanol alters *P. aeruginosa* phenazine production through a WspR-independent pathway

**Figure 8:**
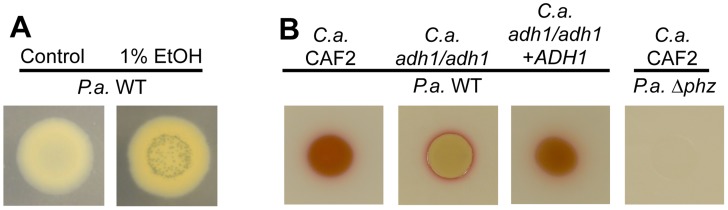
Ethanol leads to higher levels of PCN crystal formation and 5MPCA derivatives. **A**. *P. aeruginosa* strain PA14 wild type (WT) was grown on medium without and with 1% ethanol. With ethanol, PCN crystals form and the colony has a yellowish color likely attributed to reduced PCN. **B**. *P. aeruginosa* strain PA14 WT was cultured on lawns of *C. albicans* CAF2 (WT reference strain), the *C. albicans adh1/adh1* mutant, and its complemented derivative (*adh1/adh1+ADH1*); the PA14 Δ*phz* mutant defective in phenazine production was plated on the *C. albicans* CAF2 for comparison.

In part, these studies were instigated by the finding that *P. aeruginosa* phenazines strongly stimulate *C. albicans* ethanol production [18]. Thus, we were intrigued by the observation that colonies on ethanol-containing swarm plates, but not control plates, contained abundant emerald green crystals, similar to those formed by reduced phenazine-1-carboxamide (PCN) [40] (Fig. 8A, Fig. 4 and Fig. S7A), which could indicate a reciprocal relationship between ethanol and phenazines. Phenazine concentrations were measured using HPLC in either extracts from *P. aeruginosa* strain PA14 colonies or extracts from the underlying agar. In extracts from wild type colonies, PCN and PCA concentrations were 24.2- and 5.8-fold higher, respectively, when ethanol was in the medium (Fig. S7B); much smaller differences in PCN and PCA concentrations were found in extracts of the underlying agar (Fig. S7C). Because PCA is the precursor for all other phenazine derivatives, including PCN (Fig. S7A), we further explored the effect of ethanol on PCA production. For this, we measured levels of PCA in a strain lacking all of the PCA modifying enzymes (PhzH, PhzM, and PhzS; see Fig. S7A for pathways) [41]. We found that Δ*phzHMS* colonies contained 1.7-fold more PCA (Fig. S7D) and released 1.3-fold more PCA into the agar (Fig. S7E) when grown in the presence of ethanol compared to control conditions. These data suggest that ethanol may cause a minor increase in PCA, and that it has greater effects on which species of phenazines are formed. The differences in phenazine levels or profiles did not appear to be responsible for ethanol effects on swarming as the Δ*phz* mutant [42], which lacks *phzA1-G1* and *phzA2-G2*, was like the wild type in that its swarming was repressed in the presence of ethanol, but it swarmed robustly in its absence (Fig. S7F).

To determine if there was a connection between ethanol effects on Wsp signaling and ethanol stimulation of PCN levels, we assessed PCN accumulation in mutants lacking *wspR* or *pelA*. We found that both strains responded like the wild type in terms of PCN crystal formation upon growth with ethanol (Fig. S8A and Fig. 4). Similarly, ethanol catabolic mutants still showed enhanced levels of PCN crystals upon ethanol exposure (Fig. S8A).

Having observed alterations in the phenazine profile induced by ethanol, we examined the impact of ethanol in the production of a fourth phenazine derivative, 5MPCA, which we have previously shown to be released by *P. aeruginosa* when in the presence of *C. albicans* [19], [24]. Because *P. aeruginosa*-produced 5MPCA is converted into a red pigment within *C. albicans* cells, 5MPCA accumulation can be followed by observing the formation of a red color where *P. aeruginosa* and *C. albicans* are cultured together [19], [24]. To examine the effects of ethanol production on the accumulation of red 5MCPA derivatives, we again used the *C. albicans adh1*/*adh1* mutant and its complemented derivative. Strikingly, when *P. aeruginosa* was cultured on lawns of the *C. albicans adh1*/*adh1* strain, a strong decrease in red pigmentation was observed (Fig. 8B). When *ADH1* was provided in trans to the *adh1*/*adh1* mutant, accumulation of the red pigment was restored (Fig. 8B). Neither ethanol catabolism nor WspR activity was required for the stimulation of levels of 5MPCA derivatives by *P. aeruginosa* on fungal lawns (Fig. S8B).

Together, our data suggest that ethanol only slightly increases total phenazine production (Fig. S7D and E) but more strongly affects the derivatization of phenazines in *P. aeruginosa* colonies (Fig. S7B and C). Furthermore, *C. albicans*-produced ethanol stimulated *P. aeruginosa* 5MPCA production, and in turn, phenazines, including 5MPCA analogs, promote ethanol production [18]. Thus, it appears that *P. aeruginosa*-*C. albicans* interactions include a positive feedback loop that promotes fungal ethanol production and *P. aeruginosa* Wsp-dependent biofilm formation when the two species are cultured together.

## Discussion

**Figure 9:**
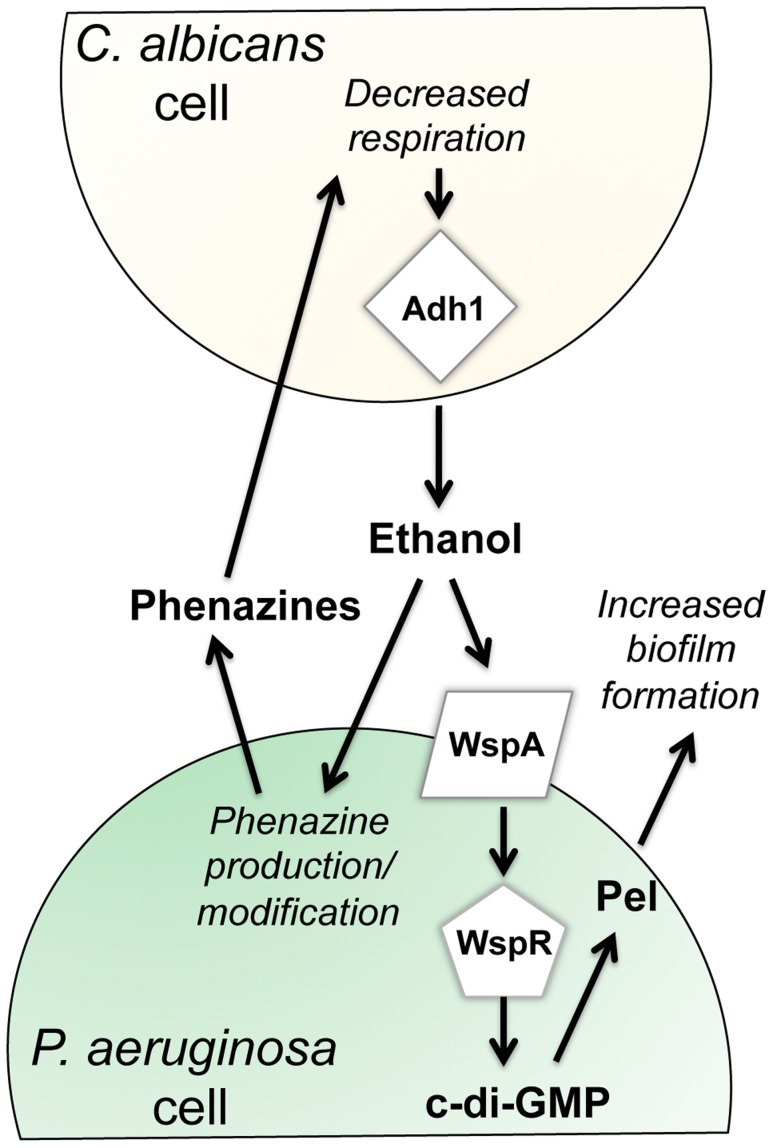
Our proposed model for the impacts of fungally-produced ethanol on *P. aeruginosa* behaviors. Our previous work has shown that *P. aeruginosa* phenazines increase fungal ethanol production. Here, we show that ethanol stimulates the Wsp system, leading to a WspR-dependent increase in c-di-GMP levels and a concomitant increase in Pel production and biofilm formation on plastic and on airway epithelial cells. In addition, ethanol altered phenazine production by promoting 5MPCA release and the accumulation of PCN.

This paper reports new effects of ethanol on *P. aeruginosa* virulence-related traits, and illustrates that these effects occur through multiple pathways (Fig. 9). We found that ethanol: i) promoted attachment to and colonization of plastic and airway epithelial cells, ii) decreased swarming, but not twitching motility, iii) increased Pel-dependent colony wrinkling, and iv) increased c-di-GMP levels. All of these responses to ethanol required the diguanylate cyclase WspR. WspR is part of the Wsp chemosensory system, which is a member of the “alternative cellular function” (ACF) chemotaxis family [20], [21], [43]. The Wsp chemosensory system is different from the chemotaxis systems in *P. aeruginosa* in terms of its localization and response to environmental signals [44]. The membrane-bound receptor WspA and the CheA homologue WspE are necessary for the Wsp system to function, and WspE activates WspR via phosphorylation [44]. Consistent with our hypothesis that the entire Wsp system is required for the response to ethanol, we found that a *wspA* mutant was also insensitive to the effects of ethanol on biofilm formation (Fig. 5). The activation of WspR was independent of ethanol catabolism and independent of phenazine production. Ethanol and other alcohols can increase the rigidity of cell membranes by promoting an altered composition of fatty acids [45], and future studies will determine if the Wsp system, particularly the membrane localized WspA, can be activated by changes in the lipid composition or changes in the physical properties of *P. aeruginosa* membranes. Because the Wsp system is also activated upon contact with a surface [20], it is intriguing to consider how these stimuli might be similar. Ethanol had mild, if any, effects, on biofilm formation at the air-liquid interface in a commonly used 96-well microtiter dish assay in either strain (Fig. S9) suggesting that in this environment, different Wsp activating cues were not additive.

*C. albicans* and other *Candida* spp. are commonly detected in the sputum of CF patients, and clinical studies suggest that the presence of both *P. aeruginosa* and *C. albicans* results in a worse prognosis for CF patients [6], [46]. *In vivo* ethanol production by other fungi has been documented [47], [48], but a link between *Candida* spp. and ethanol production in the lung has not yet been made. It is important to note, however, that ethanol was one of two metabolites in exhaled breath condensate that differentiated CF from non-CF individuals [49]. Thus, regardless of the source of ethanol, be it fungal or bacterial, the effect of ethanol on pathogens such as *P. aeruginosa* is likely of biological and clinical relevance. We tested this interaction in the context of CF, but this polymicrobial interaction likely occurs in other contexts as well.

As shown above, ethanol promoted biofilm formation and likely concomitant increases in drug tolerance. In the airway epithelial cell system, *P. aeruginosa* CFU recovery was increased 3-fold by addition of ethanol (Fig. 6B) and 4.7-fold by co-culture with *C. albicans* (Fig. 7). A two-fold difference is comparable to the differences in colonization between wild-type *P. aeruginosa* strains and mutants lacking genes known to play a role in virulence in animal models. For example, a Δ*plcHR* mutant lacking hemolytic phospholipase C or a Δ*anr* strain defective in a global regulator have 1.3- to 2.6-fold fewer CFUs recovered from airway cells compared to wild type, and notable differences in animal models [50], [51]. Hence the presence of ethanol may result in increased virulence of *P. aeruginosa* in the host. Ethanol has also been shown to promote *P. aeruginosa* conversion to a mucoid state [52], in which the exopolysaccharide alginate is overproduced; mucoidy is common in CF isolates and is correlated with a decline in lung function [53], [54]. Ethanol has been shown to enhance virulence and biofilm formation by other lung pathogens such as *Staphylococcus aureus* [55] and *Acinetobacter baumanii* [56]–[59] via mechanisms that have not yet been described. Like in *P. aeruginosa* (Fig. S1A), ethanol caused a slight stimulation of growth in *A. baumanii* [58].

In addition to the effects of ethanol on *P. aeruginosa*, ethanol is an immunosuppressant that negatively influences the lung immune response [60]–[64]. In a mouse model, ethanol inhibits lung clearance of *P. aeruginosa* by inhibiting macrophage recruitment [65]. Together, these observations suggest that in mixed infections, *P. aeruginosa* may promote the production of ethanol by fungi, and that fungally-produced ethanol may in turn enhance the virulence and persistence of co-existing pathogens, and thus may directly impact the host.

It is not yet known how ethanol influences the spectrum of *P. aeruginosa* phenazines produced. In a previous study, we found evidence for increased production and release of 5MPCA when *P. aeruginosa* is grown in co-culture with *C. albicans*, and that live *C. albicans* is required for this effect [19]. More recent studies show that *C. albicans* ethanol production increased in the presence of even very low concentrations of the 5MPCA analog phenazine methosulfate [18], that the 5MPCA-like compounds were even more effective inhibitors of fungi than PCA and PYO, the two phenazines normally produced when *P. aeruginosa* is grown in mono-culture. Here, our findings suggest a feedback loop in which *C. albicans*-produced ethanol promoted the release of phenazines (Fig. 7) that may promote further ethanol production [18]. It is also important to consider that some studies have reported that 5MPCA and PCN have enhanced antifungal activity when compared to PCA and PYO [19], [23], [24]. The ethanol-induced changes in PCA were not as dramatic when compared to the ethanol-induced changes in PCN and 5MPCA, suggesting that ethanol mainly affected the biosynthetic steps after the formation of PCA leading to its conversion to PCN, 5MPCA and PYO. In different settings, such as liquid cultures or in clinical isolates lacking activity of LasR, a transcriptional regulator for quorum sensing that controls phenazine production, the presence of *C. albicans* enhanced the production of 5MPCA and PYO [24], [25]. Taken together, all these observations indicate that fungally-produced ethanol may enhance the conversion of PCA to end products such as PCN, 5MPCA and PYO.

These studies indicate how microbial species can alter the behavior of one another and suggest that the nature of these dynamic interactions can change depending on the context. In the rhizosphere, where pseudomonad antagonism of fungi includes the colonization of fungal hyphae and phenazine production, the enhancement of fungally-produced ethanol by phenazines and stimulation of biofilm formation and phenazine production by ethanol may create a cycle that is relevant to biocontrol [23], [66], [67]. In chronic infections where these two species are found together, such as in chronic CF-associated lung disease, this molecular interplay may be synergistic and promote long-term colonization of both species in the host. These findings indicate that the treatment of colonizing fungi may be beneficial due to their effects on other pathogens even if the fungi themselves are not acting as overt agents of host damage.

## Materials and Methods

### Strains, media, and growth conditions

Bacterial and fungal strains and plasmids used in this study are listed in Table S1. Bacteria and fungi were maintained on LB [68] and YPD (2% peptone, 1% yeast extract, and 2% glucose) media, respectively. When stated, ethanol (200-proof), choline chloride or glycerol was added to the medium (liquid or molten agar) to a final concentration of 1%. Control cultures received an equivalent volume of water. When ethanol was supplied as a sole carbon source, glucose and amino acids were omitted. Mutants from the PA14 Non-Redundant (NR) Library were grown on LB with 30 µg/mL gentamicin [29]. When strains for the NR library were used, the location of the transposon insertion was confirmed using sets of site-specific primers followed by sequencing of the amplicon. The primers are listed in Table S2.

### Growth curve analysis of *P. aeruginosa* in the presence of ethanol

For growth curves, overnight cultures were diluted into 5 ml fresh medium (LB or M63 with 0.2% glucose [69] with or without ethanol) to an OD_600 nm_ of ∼0.05 and incubated at 37°C on a roller drum. Culture densities below 1.5 were measured directly in the culture tubes using a Spectronic 20 spectrophotometer. At higher cell densities, diluted culture aliquots were measured using a Genesys 6 spectrophotometer.

### Quantification of *P. aeruginosa* attachment to plastic and airway epithelial cells

To measure the attachment of cells to the plastic surface in 6-well or 12-well untreated polystyrene plates, wells were inoculated with a suspension of cells at an initial OD_600 nm_ of 0.002 from overnight cultures. Every 90 minutes, the culture medium was removed and fresh medium was supplied. Pictures were taken using an inverted Zeiss Axiovert 200 microscope with a long distance 63× DIC objective at specified intervals. To quantify the number of cells or microcolonies in control cultures compared to cultures with ethanol, images were captured, randomized, and analyzed by a researcher who was blind to the identity of the sample at the time of analysis. In each experiment, more than 10 fields were counted for each strain. Microcolonies were defined as clusters of more than 5 cells in physical contact with one another. Biofilm formation on plastic microtiter dishes were performed and analyzed using the crystal violet assay as described in [55] and biofilm values were measured by quantification of dye as measured absorbance at 650 nm.

The analysis of *P. aeruginosa* colonization of airway epithelial cells was performed using CFBE human bronchial epithelial cells (CFBE410^−^) with the *CFTR*ΔF508/ΔF508 genotype [70] as described previously [35], [36]. For imaging, cells were grown in 6-well glass bottom dishes (MatTek). For quantification of attached cells, CFBEs were grown in 6 or 12 well plates. *P. aeruginosa* strain PAO1 cells were added at an MOI of 30∶1, and the medium was exchanged every 1.5 hours. For experiments with *C. albicans*, PAO1 cells and *C.albicans* were added together to CFBE monolayers, where *C.albicans* was at an MOI of 10∶1 with respect to the epithelial cells. Pictures were taken using a Zeiss Axiovert 200 microscope with a 63× DIC objective at specified intervals. We performed multiple experiments with technical replicates (between three and six) on different days and analyzed the data with a one-way analysis of variance and Tukey's post hoc *t*-test using Graph Pad Prism 6. We observed that cells from different passages had differences in the mean attachment across all samples from that day. Thus, we normalized values to the mean across all samples from each experiment. LDH release was measured after six hours using the Promega CytoTox96 Non-Radioactive Cytotoxicity kit as described in the manufacturer's instructions.

### Analysis of *P. aeruginosa* swarming and twitching motility

Swarming motility was tested by inoculating 2.5 µL of overnight cultures on fresh M8 (M8 salts without trace elements supplemented with 0.2% glucose, 0.5% casamino acids, and 1 µM MgSO_4_) containing 0.5% agar as described previously [71]. Plates were incubated face up at 37°C with 70–80% humidity in stacks of no more than 4 for 16.5 hrs. To quantify the degree of swarming, percent coverage of the plate was measured using ImageJ software [72]. Twitching motility was analyzed as described previously [26].

### Cyclic-di-GMP measurements

Cells were collected from swarm plates after incubation at 37°C for 16.5 h and placed in pre-weighed 1.5 mL Eppendorf tubes. Tubes were centrifuged at 5,000 rpm for 4 minutes. The pellets were then resuspended in 250 µL of extraction buffer by vigorous vortexing (extraction buffer: MeOH/acetonitrile/dH_2_O 40∶40∶20+0.1 N formic acid stored at −20°C). The extractions were incubated at −20°C for 30 minutes in an upright position. The tubes were then centrifuged at 13,000 rpm for 5 minutes at 4°C. 200 µL of the extraction were recovered into new Eppendorf tubes and neutralized with 4 µL of 15% NH_4_HCO_3_ per 100 µL of sample. The tubes with cell debris were left to dry and reweighed for normalization of cell numbers from swarm plates. 150 µL of samples were sent to the RTSF Mass Spectrometry and Metabolomics Core at Michigan State University for LC-MS analysis.

### Microscopic analysis of WspR

Sample preparation and microscopy were performed as previously described [20], [34]. To analyze liquid-grown cells, cultures were grown at 37°C while shaking to an optical density at 600 nm (OD_600_) of 0.3 in M9 medium (1× M9 salts pH 7.4, 2 mM MgSO_4_, 0.1 mM CaCl_2_, 0.2% glycerol, 0.2% casamino acids and 10 µg/ml thiamine HCl). 1% arabinose was included for induction of *wspR*, and 1% ethanol was added when comparing its effect on WspR clustering. From each culture, 3 µl were spotted onto a 0.8% agarose PBS pad on a microscope slide and then covered with a coverslip.

More than 100 cells were counted for each condition.

### *P. aeruginosa-C. albicans* co-cultures

Preformed lawns of *C. albicans* CAF2 and *adh1/adh1* were prepared by spreading 700 µL of a YPD-grown overnight culture onto a YPD 1.5% agar plate followed by incubation at 30°C for 48 hr. Exponential phase *P. aeruginosa* liquid cultures were spotted (5–10 µL) onto the *C. albicans* lawns, then incubated at 30°C for an additional 24 to 72 hours.

### Analysis of phenazines

Overnight cultures of *P. aeruginosa* PA14 wild-type and Δ*phzH*Δ*phzM*Δ*phzS* strains were grown in LB at 37°C (shaken at 250 rpm). Ten microliters of each culture were spotted onto a track-etched membrane (Whatman 110606; pore size 0.2 µm; diameter 2.5 cm) that was placed on a 1.5% agar M8 medium supplemented with either vehicle (water) or 1% v/v ethanol. Plates contained 3 ml of medium in a 35×10 mm agar plate (Falcon). The colonies were incubated at 37°C for 24 hours and then at room temperature for 72 hours, after which phenazines were extracted from the colonies and agar separately. Each track-etched membrane with a colony was lifted off the agar plates and nutated in 5 mL of 100% methanol overnight at room temperature. Similarly, the agar was nutated overnight in 5 mL of 100% methanol. Colony and agar extracts were filtered (0.2 µm pore) and phenazines in the extraction volume (5 mL) were quantified by high-performance liquid chromatography as previously described [41] at a flow rate of 0.4 mL/min.

### Statistical analyses

All data were analyzed using Graph Pad Prism 6. The data represent the mean standard deviation of at least three independent experiments with multiple replicates unless stated otherwise. For normally distributed data, comparisons were tested with Student's t-test.

## Supporting Information

Figure S1 **Ethanol does not affect** ***P. aeruginosa*** **PA14 WT growth.** Growth kinetics in M63 medium with 0.2% (w/v) glucose and 0.5% (w/v) casamino acids with and without 1% ethanol (EtOH). Error bars represent one standard deviation; N = 3). Colony growth on the same medium with 1.5% agar with or without 1% ethanol is also shown (inset).
(TIF)

Figure S2 **Ethanol does not inhibit twitching behavior in** ***P. aeruginosa*** **.** Twitching motility in *P. aeruginosa* strain PA14 wild type and Δ*pilA* (a strain defective in twitching motility) in the absence and presence of 1% ethanol (EtOH). Average twitch diameters are 16 mm in controls and 17 mm with ethanol (N = 16).
(TIF)

Figure S3 **Ethanol catabolism is not required for the suppression of swarming.** **A**. PA14 *exaA*::Tn*M*, PA14 *pqqB*::Tn*M*, and PA14 *acsA*::Tn*M* grew on glucose, but were unable to utilize ethanol (EtOH) as a sole source of carbon. **B**. Swarm analysis of PA14 WT and ethanol catabolism mutants (*exaA*::Tn*M*, *pqqB*::Tn*M*, and *acsA*::Tn*M*) in the absence and presence of ethanol. Pictures were taken at 16.5 h after inoculation and are representative of at least 3 separate experiments. **C**. Quantification of percent coverage of swarm plates for the strains shown in B. Error bars represent one standard deviation among five replicates within one experiment.
(TIF)

Figure S4 **Effect of other carbon sources found in the CF lung on swarming motility.** Swarming by *P. aeruginosa* strain PA14 WT was assessed on swarm medium amended with vehicle alone (control), 1% choline or 1% glycerol. Pictures were captured at 16.5 h and are representative of at least 3 separate experiments.
(TIF)

Figure S5 **Wrinkling is enhanced by 1% ethanol in both strains PA14 and PAO1.** *P. aeruginosa* strain PA14 *flgK*::Tn*5* and PAO1 *flgK*::Tn*5* spot inoculated colonies on swarm agar without (control) and with 1% ethanol (EtOH) after 72 h.
(TIF)

Figure S6 ***Candida albicans*** **does not lead to ethanol-dependent increases in colonization of airway epithelial cells in the Δ** ***wspR*** **and Δ** ***wspA*** **backgrounds.** *P. aeruginosa* PAO1 Δ*wspR* and Δ*wspA* were cultured with a monolayer of ΔF508 CFTR-CFBE cells and either the *C. albicans* CAF2 (WT reference strain) or the *C. albicans adh1/adh1* mutant (*adh1*). Data represent the average of three technical replicates per experiment and the experiment was performed twice. Error bars represent the standard deviation among replicates.
(TIF)

Figure S7 **Ethanol stimulates PCN production but not PCA production in** ***P. aeruginosa*** **strain PA14.** **A**. Phenazine biosynthetic pathway and enzymes necessary for phenazine modifications. **B–E**. Concentrations of PCN and PCA in 5 ml extracts from the colony (**B**) or the underlying agar (**C**). In **B** and **C**, the wild type (WT) was grown without and with 1% ethanol. In **D** and **E**, PA14 Δ*phzHMS*, which lacks the ability to transform PCA into phenazine derivatives, was used. The error bars represent standard deviations for the phenazines extracted from 6 samples; *, *P*>0.05; **, *P*≤0.05; ***, *P*≤0.01, ****, *P*≤0.001; ns, *P*>0.05. **F**. Swarm phenotype of the Δ*phz* mutant without and with 1% ethanol.
(TIF)

Figure S8 **Neither** ***wspR*** **nor ethanol catabolism are solely responsible for increased PCN or 5MPCA.** **A**. Spot colonies of *P. aeruginosa* strain PA14 ethanol catabolism mutants and the Δ*wspR* strain were grown in the absence and presence of 1% ethanol for 8 days, then imaged. **B**. *C. albicans* CAF2 (wild type) lawns were spot inoculated with *P. aeruginosa* strain PA14 wild type (WT), Δ*wspR*, or *exaA*::Tn*M*, and incubated at 30°C for 24 h, then at room temperature for 36 h.
(TIF)

Figure S9 **Ethanol has modest, if any, effects on biofilm formation in a microtiter dish assay.** *P. aeruginosa* strain PAO1 and PA14 were grown in M63 medium with glucose and casamino acids either without or with 1% ethanol (EtOH). While strain PAO1 showed modest stimulation at 24 h, strain PA14 did not show stimulation of biofilm at this time point. Biofilms were measured by crystal violet staining followed by solubilization and measured as absorbance at 650 nm. Differences between control and with ethanol were small but significant and reproducible (p<0.05) for strain PAO1 and not significant for strain PA14.
(TIF)

Table S1 **Strain and plasmid list.**
(DOCX)

Table S2 **Primers used in this study.**
(DOCX)
