# Supplementary figures and images for: Candida albicans Ethanol Stimulates Pseudomonas aeruginosa WspR-Controlled Biofilm Formation as Part of a Cyclic Relationship Involving Phenazines
Source: PLoS Pathog. 2014 Oct 23;10(10):e1004480. doi: 10.1371/journal.ppat.1004480 (PMC4207824; doi:10.1371/journal.ppat.1004480)

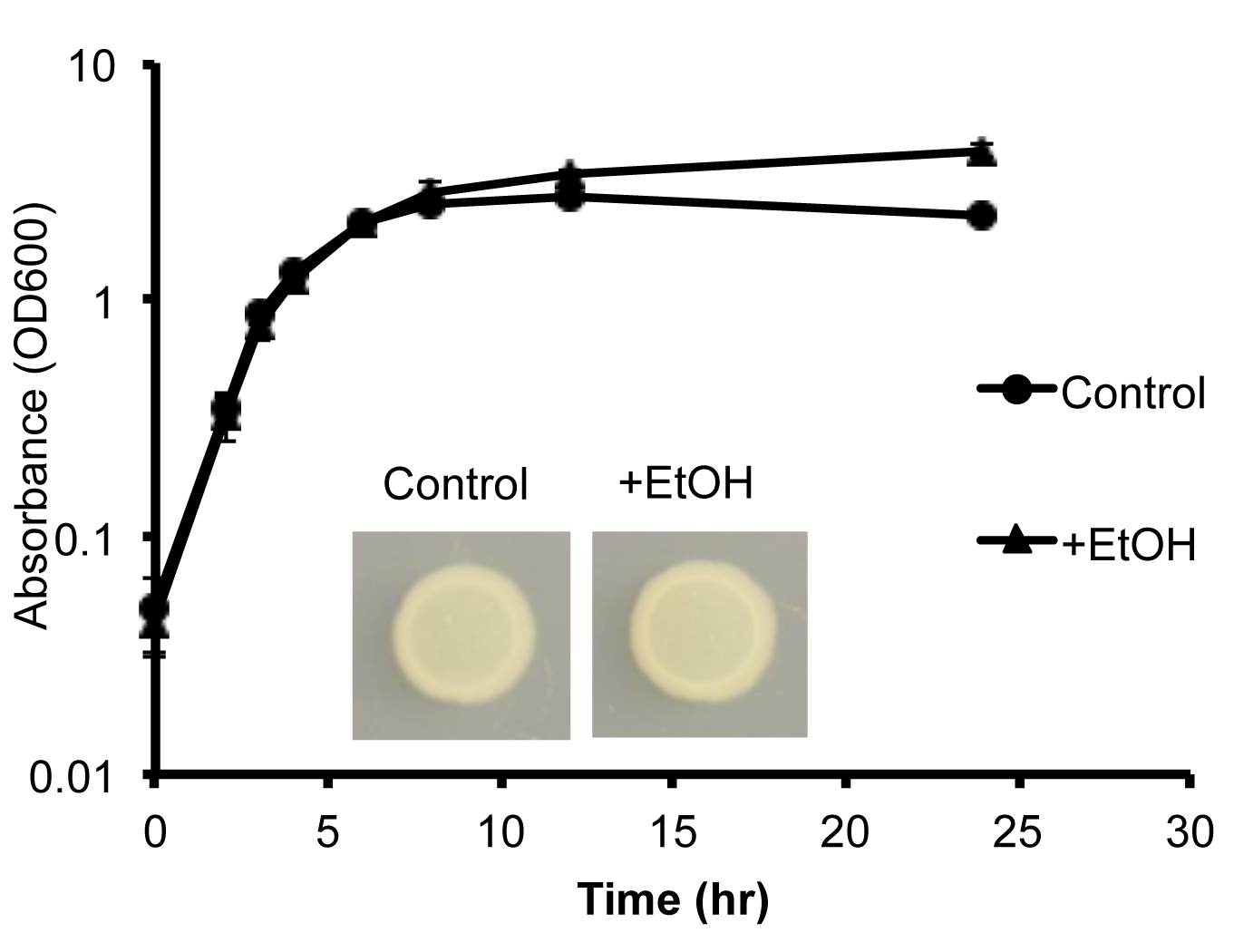

Supplement: Figure S1 — Ethanol does not affect P. aeruginosa PA14 WT growth. Growth kinetics in M63 medium with 0.2% (w/v) glucose and 0.5% (w/v) casamino acids with and without 1% ethanol (EtOH). Error bars represent one standard deviation; N = 3). Colony growth on the same medium with 1.5% agar with or without 1% ethanol is also shown (inset). (TIF) [file ppat.1004480.s001.tif]

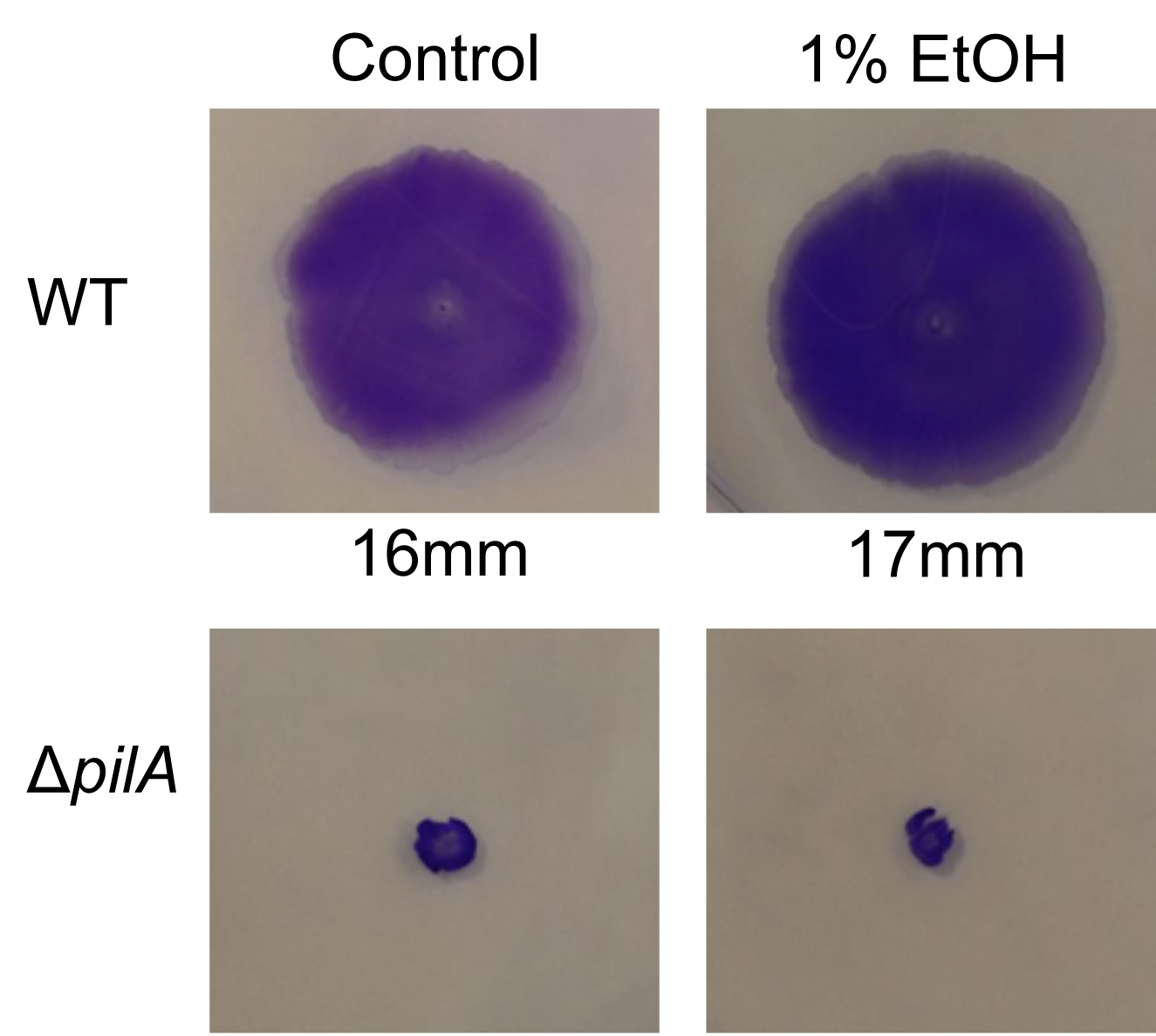

Supplement: Figure S2 — Ethanol does not inhibit twitching behavior in P. aeruginosa . Twitching motility in P. aeruginosa strain PA14 wild type and ΔpilA (a strain defective in twitching motility) in the absence and presence of 1% ethanol (EtOH). Average twitch diameters are 16 mm in controls and 17 mm with ethanol (N = 16). (TIF) [file ppat.1004480.s002.tif]

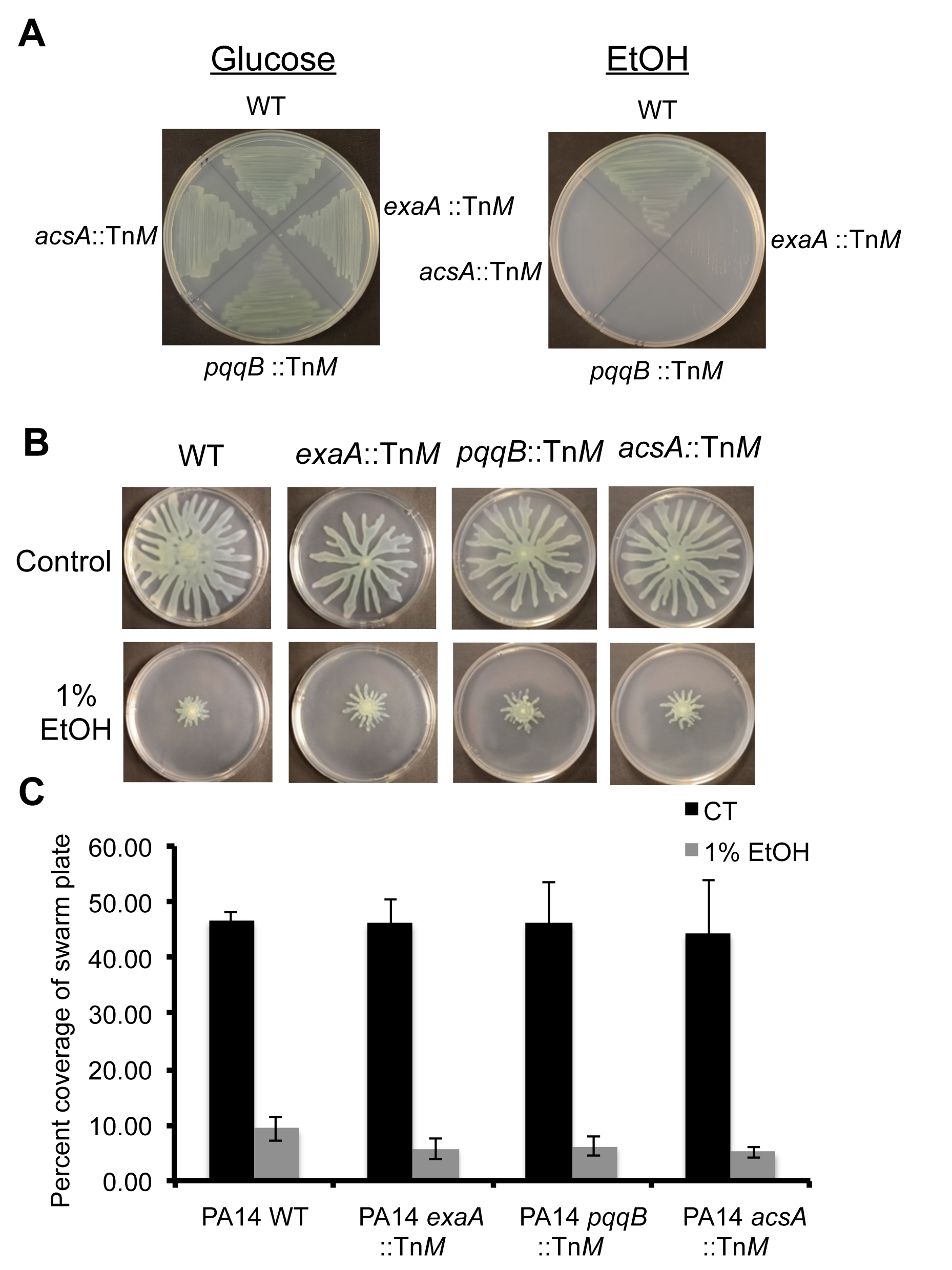

Supplement: Figure S3 — Ethanol catabolism is not required for the suppression of swarming. A. PA14 exaA::TnM, PA14 pqqB::TnM, and PA14 acsA::TnM grew on glucose, but were unable to utilize ethanol (EtOH) as a sole source of carbon. B. Swarm analysis of PA14 WT and ethanol catabolism mutants (exaA::TnM, pqqB::TnM, and acsA::TnM) in the absence and presence of ethanol. Pictures were taken at 16.5 h after inoculation and are representative of at least 3 separate experiments. C. Quantification of percent coverage of swarm plates for the strains shown in B. Error bars represent one standard deviation among five replicates within one experiment. (TIF) [file ppat.1004480.s003.tif]

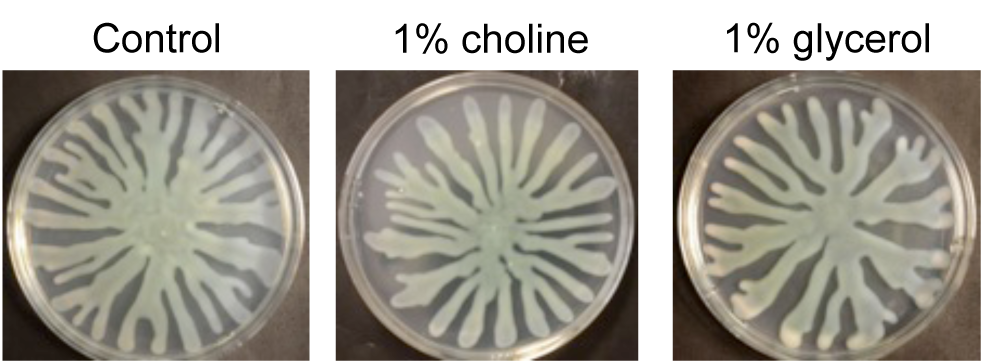

Supplement: Figure S4 — Effect of other carbon sources found in the CF lung on swarming motility. Swarming by P. aeruginosa strain PA14 WT was assessed on swarm medium amended with vehicle alone (control), 1% choline or 1% glycerol. Pictures were captured at 16.5 h and are representative of at least 3 separate experiments. (TIF) [file ppat.1004480.s004.tif]

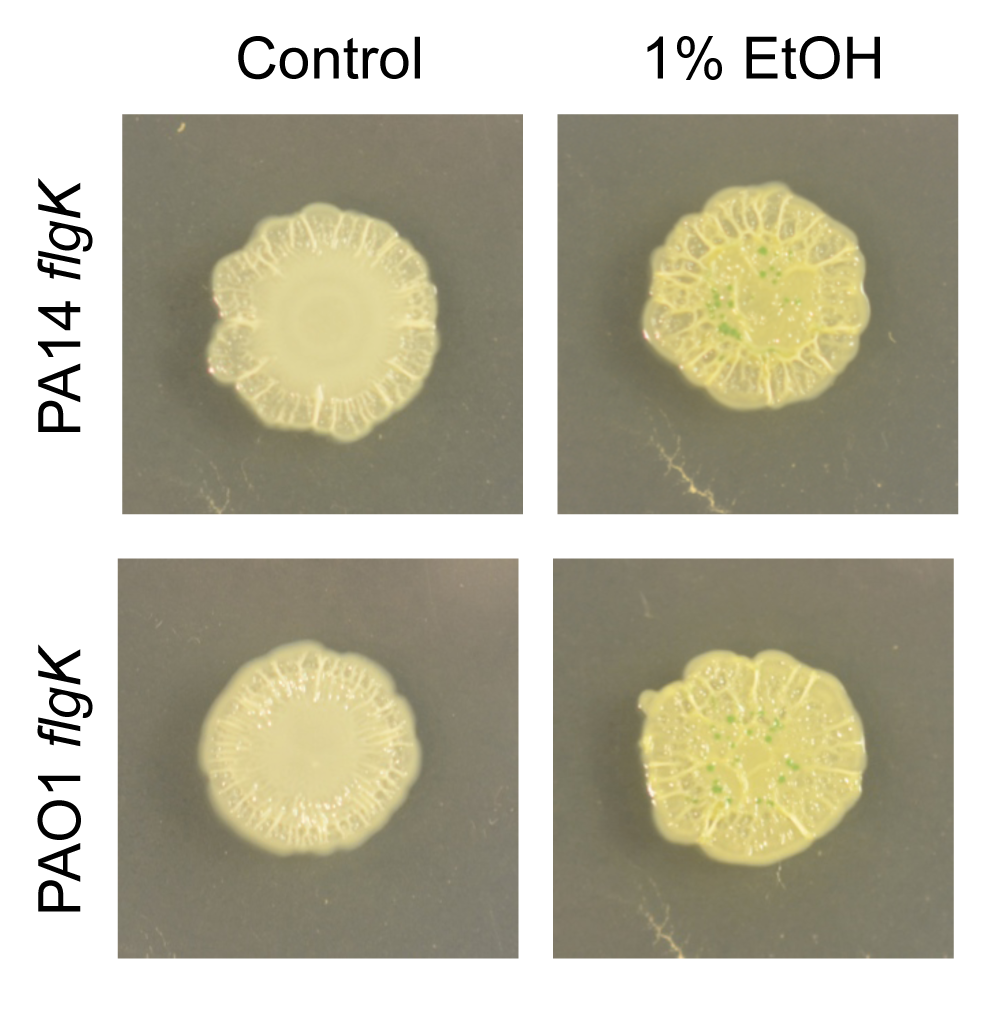

Supplement: Figure S5 — Wrinkling is enhanced by 1% ethanol in both strains PA14 and PAO1. P. aeruginosa strain PA14 flgK::Tn5 and PAO1 flgK::Tn5 spot inoculated colonies on swarm agar without (control) and with 1% ethanol (EtOH) after 72 h. (TIF) [file ppat.1004480.s005.tif]

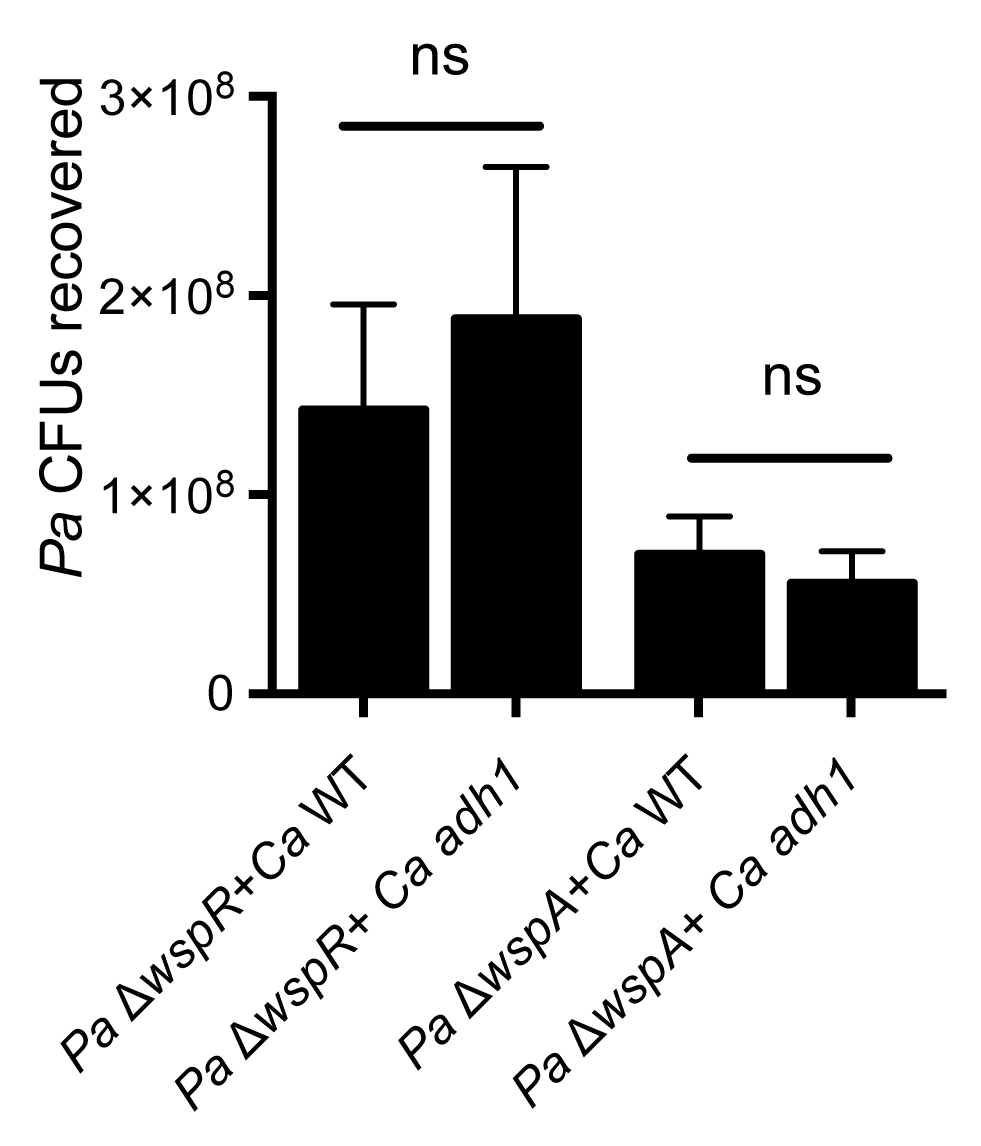

Supplement: Figure S6 — Candida albicans does not lead to ethanol-dependent increases in colonization of airway epithelial cells in the Δ wspR and Δ wspA backgrounds. P. aeruginosa PAO1 ΔwspR and ΔwspA were cultured with a monolayer of ΔF508 CFTR-CFBE cells and either the C. albicans CAF2 (WT reference strain) or the C. albicans adh1/adh1 mutant (adh1). Data represent the average of three technical replicates per experiment and the experiment was performed twice. Error bars represent the standard deviation among replicates. (TIF) [file ppat.1004480.s006.tif]

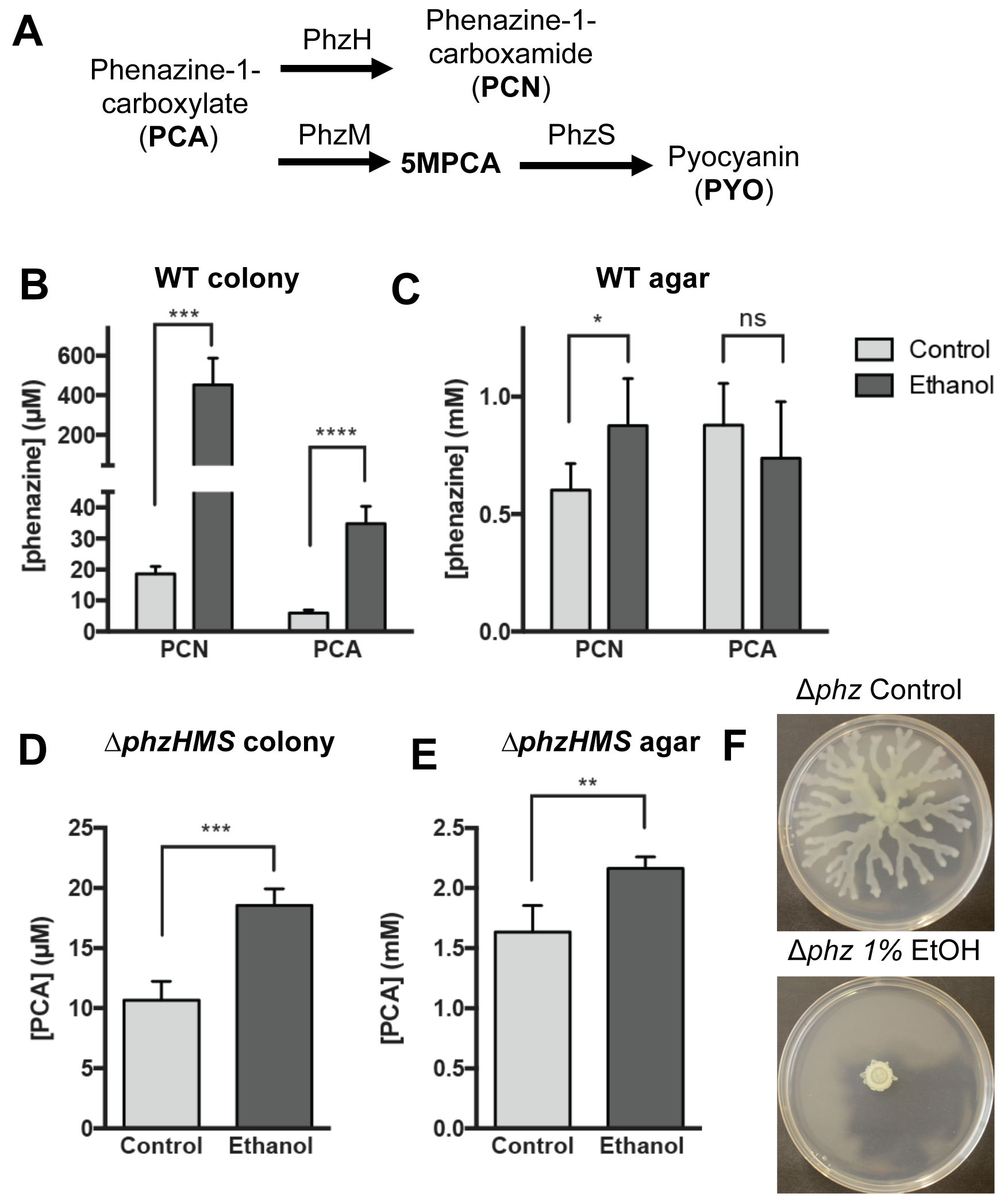

Supplement: Figure S7 — Ethanol stimulates PCN production but not PCA production in P. aeruginosa strain PA14. A. Phenazine biosynthetic pathway and enzymes necessary for phenazine modifications. B–E. Concentrations of PCN and PCA in 5 ml extracts from the colony (B) or the underlying agar (C). In B and C, the wild type (WT) was grown without and with 1% ethanol. In D and E, PA14 ΔphzHMS, which lacks the ability to transform PCA into phenazine derivatives, was used. The error bars represent standard deviations for the phenazines extracted from 6 samples; *, P>0.05; **, P≤0.05; ***, P≤0.01, ****, P≤0.001; ns, P>0.05. F. Swarm phenotype of the Δphz mutant without and with 1% ethanol. (TIF) [file ppat.1004480.s007.tif]

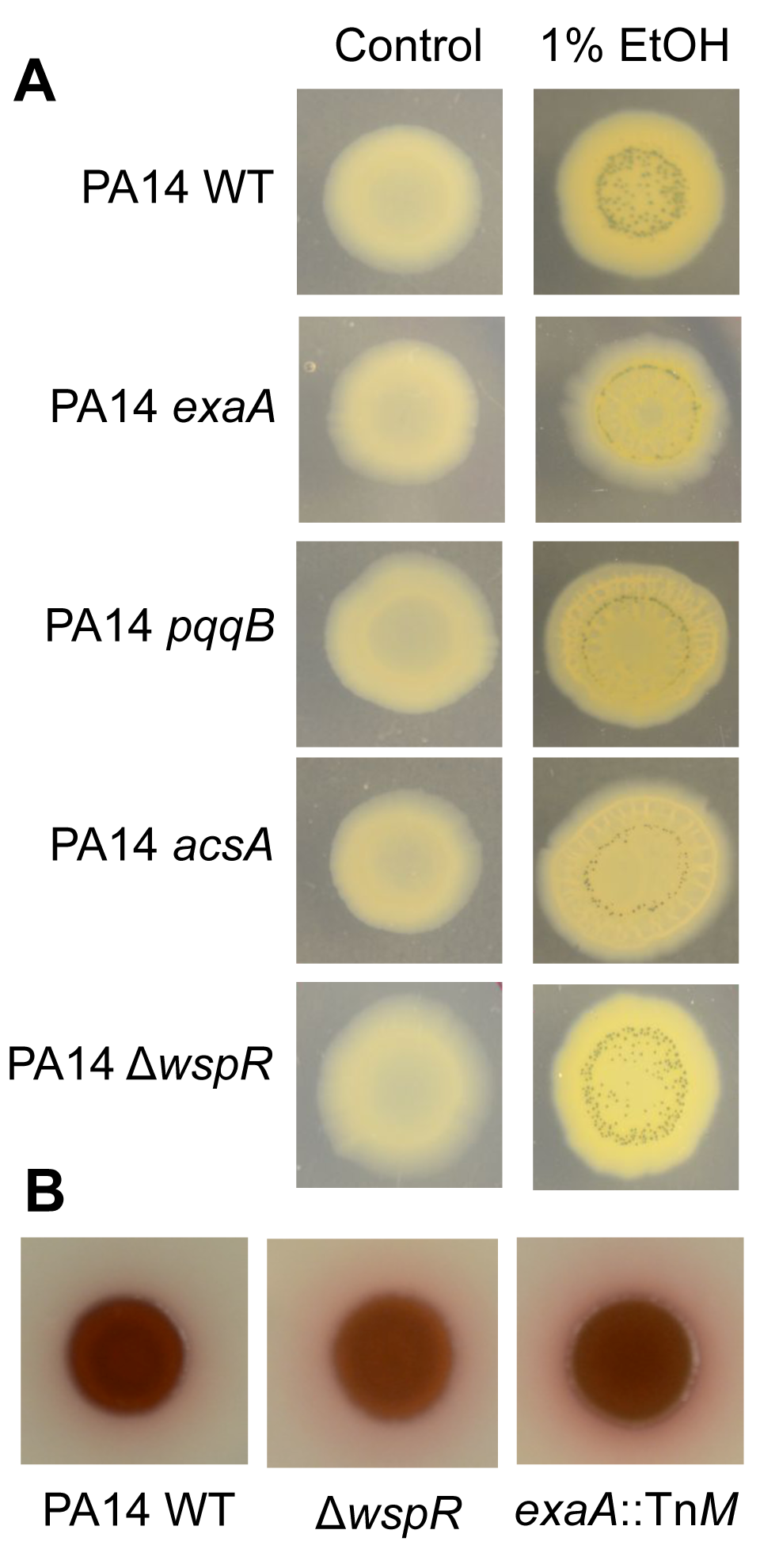

Supplement: Figure S8 — Neither wspR nor ethanol catabolism are solely responsible for increased PCN or 5MPCA. A. Spot colonies of P. aeruginosa strain PA14 ethanol catabolism mutants and the ΔwspR strain were grown in the absence and presence of 1% ethanol for 8 days, then imaged. B. C. albicans CAF2 (wild type) lawns were spot inoculated with P. aeruginosa strain PA14 wild type (WT), ΔwspR, or exaA::TnM, and incubated at 30°C for 24 h, then at room temperature for 36 h. (TIF) [file ppat.1004480.s008.tif]

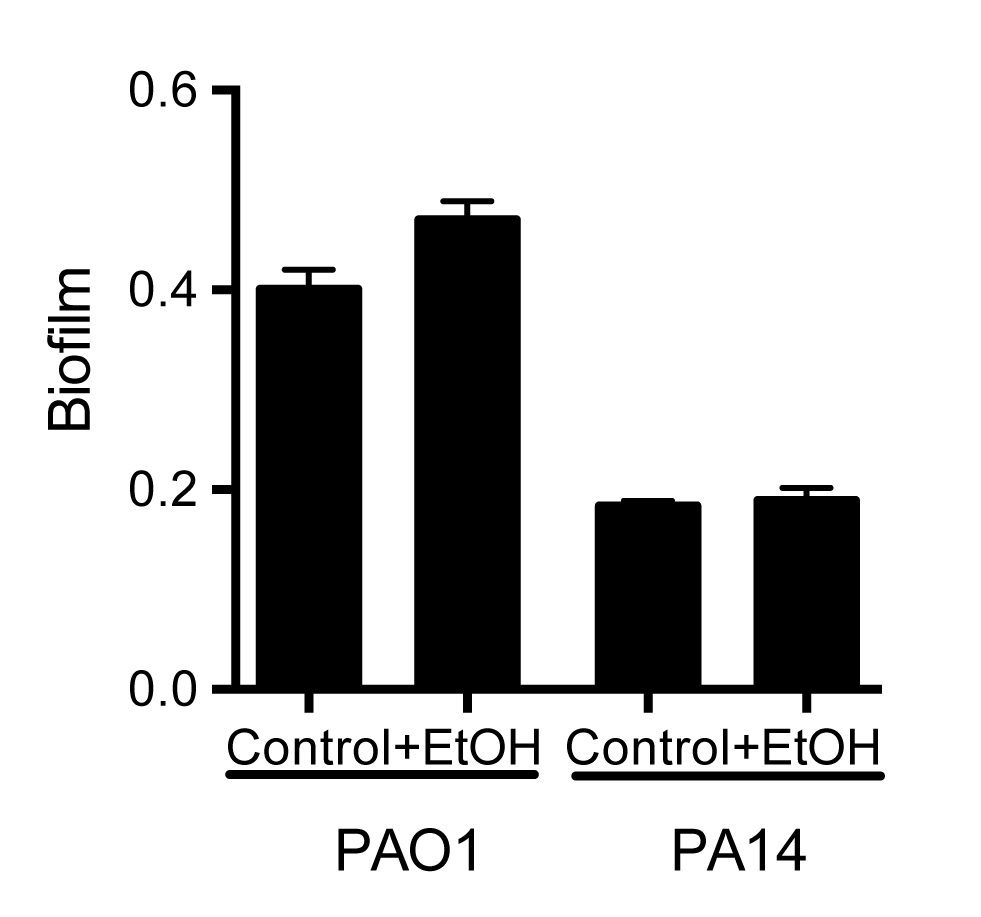

Supplement: Figure S9 — Ethanol has modest, if any, effects on biofilm formation in a microtiter dish assay. P. aeruginosa strain PAO1 and PA14 were grown in M63 medium with glucose and casamino acids either without or with 1% ethanol (EtOH). While strain PAO1 showed modest stimulation at 24 h, strain PA14 did not show stimulation of biofilm at this time point. Biofilms were measured by crystal violet staining followed by solubilization and measured as absorbance at 650 nm. Differences between control and with ethanol were small but significant and reproducible (p<0.05) for strain PAO1 and not significant for strain PA14. (TIF) [file ppat.1004480.s009.tif]
