# Supplementary material for: Candida albicans Ethanol Stimulates Pseudomonas aeruginosa WspR-Controlled Biofilm Formation as Part of a Cyclic Relationship Involving Phenazines
Source: PLoS Pathog. 2014 Oct 23;10(10):e1004480. doi: 10.1371/journal.ppat.1004480 (PMC4207824; doi:10.1371/journal.ppat.1004480)
Supplement: Table S1 — Strain and plasmid list. (DOCX) [file ppat.1004480.s010.docx]

**Table S1.** Strain and plasmid list

| **Strains** | Lab # | **Description** | **Source** |
| --- | --- | --- | --- |
| ***P. aeruginosa*** |  |  |  |
| PA14 wild type | DH123 | Wild-type | [[70](#_ENREF_70)] |
| PA14 *flgK*::Tn*5* | DH2 | Deficient in swarming and swimming | [[32](#_ENREF_32)] |
| PA14 Δ*pilA* SMC3782 | DH2142 | Deficient in twitching |  |
| PA14 Δ*phz* | DH933 | In-frame deletions of *phzA1*-*phzG1* and *phzA2*-*phzG2* | [[45](#_ENREF_45)] |
| PA14 Δ*phzHMS* |  | PA14 with deletions of the *phzM, phzH and phzS* genes | [[34](#_ENREF_34)] |
| PA14 Δ*pelA* | DH97 | In-frame deletion mutant of *pelA* | [[41](#_ENREF_41)] |
| PA14 *exaA*::Tn*M* | DH2130 | Tn*M* mutant, lacks ethanol dehydrogenase | [[25](#_ENREF_25)] |
| PA14 *pqqB*::Tn*M* | DH2131 | Tn*M* mutant, PqqB^-^ | [[25](#_ENREF_25)] |
| PA14 *acsA*::Tn*M* | DH2132 | Tn*M*, lacks acetaldehyde dehydrogenase | [[25](#_ENREF_25)] |
| PA14 Δ*wspR* | DH2125 | In-frame deletion mutant of *wspR* | [40] |
| PA14 Δ*wspR*+*wspR* | DH2144 | DH2125 with arabinose-inducible *wspR* on plasmid pDPM73 | This study |
| PA14 Δ*wspR*+EV | DH2143 | DH2125 containing the empty vector pDPM73 | This study |
| PAO1 wild type | DH1856 | From lab of S. Dove | [[71](#_ENREF_71)] |
| PAO1 *flgK*::Tn*5* | DH2136 | From O’Toole Lab |  |
| PAO1 Δ*wspR* | PAO1103/  DH2117 | In-frame deletion mutant | [[21](#_ENREF_21)] |
| PAO1 Δ*wspA* | PAO1101/  DH2118 | In-frame deletion mutant | [[21](#_ENREF_21)] |
| PAO1 Δ*wspR attB::miniCTX-wspR^E253A^-yfp* |  | In-frame deletion of *wspR* with *wspR^E235A^-yfp* | [[33](#_ENREF_33)] |
| PAO1 Δ*wspFR attB::miniCTX-wspR^E253A^-yfp* |  | In-frame deletion of *wspF* and *wspR* with *wspR^E235A^-yfp* | [[33](#_ENREF_33)] |
| ***C. albicans*** |  |  |  |
| *C. albicans* CAF2 | DH331 | Reference strain *URA3/ura3* | [[72](#_ENREF_72)] |
| *C. albicans adh1/adh1* | DH2176 | *adh1/adh1 URA3/ura3* | This study |
| *C. albicans adh1/adh1*+*ADH1* | DH2177 | *adh1/adh1 +ADH1* | This study |
