# Supplementary material for: Candida albicans Ethanol Stimulates Pseudomonas aeruginosa WspR-Controlled Biofilm Formation as Part of a Cyclic Relationship Involving Phenazines
Source: PLoS Pathog. 2014 Oct 23;10(10):e1004480. doi: 10.1371/journal.ppat.1004480 (PMC4207824; doi:10.1371/journal.ppat.1004480)
Supplement: Table S2 — Primers used in this study. (DOCX) [file ppat.1004480.s011.docx]

Table S2. Primers used in this study

| Primer | Primer no. | Description |
| --- | --- | --- |
| R2Tn*M* | P1043 | 5’ TGTCAACTGGGTTCGTGCCTTCATCCG 3’ |
| exaA Tn conf_FWD | 558 | 5’ ACA ACG TGT TCA AGC TGA C 3’ |
| exaA Tn conf_REV | 559 | 5’ ACA CCT TGT CGC CAT AGA 3’ |
| pqqB Tn conf_FWD | 560 | 5’ GGT TTC GAA GTG ACC CTC TAC 3’ |
| pqqB Tn conf_REV | 561 | 5’ CAG GGC GAT GGA GGA TTG 3’ |
| acsA Tn conf_FWD | 562 | 5’ CGA CCA CCA GGA AAT CAC 3’ |
| acsA Tn conf_REV | 563 | 5’ GAT CAC CAC CTT CGA CTT G 3’ |
